# Loss of GATA2 promotes invasion and predicts cancer recurrence and survival in uterine serous carcinoma

**DOI:** 10.1172/jci.insight.187073

**Published:** 2025-04-01

**Authors:** Usha S. Polaki, Trey E. Gilpin, Apoorva T. Patil, Emily Chiu, Ruth Baker, Peng Liu, Tatiana S. Pavletich, Morteza Seifi, Paula M. Mañán-Mejías, Jordan Morrissey, Jenna Port, Rene Welch Schwartz, Irene M. Ong, Dina El-Rayes, Mahmoud A. Khalifa, Pei Hui, Vanessa L. Horner, María Virumbrales-Muñoz, Britt K. Erickson, Lisa Barroilhet, Stephanie M. McGregor, Emery H. Bresnick, Daniel R. Matson

**Affiliations:** 1 Department of Pathology and Laboratory Medicine and; 2 Department of Obstetrics and Gynecology, University of Wisconsin-Madison, Madison, Wisconsin USA.; 3 Department of Obstetrics, Gynecology, and Women’s Health, University of Minnesota, Minneapolis, Minnesota, USA.; 4 Department of Biostatistics and Medical Informatics and; 5 Carbone Cancer Center, University of Wisconsin–Madison, Madison, Wisconsin, USA.; 6 Wisconsin State Laboratory of Hygiene, University of Wisconsin School of Medicine and Public Health, Madison, Wisconsin, USA.; 7 Department of Cell and Regenerative Biology, University of Wisconsin–Madison, Madison, Wisconsin, USA.; 8 Department of Laboratory Medicine and Pathology, University of Minnesota, Minneapolis, Minnesota, USA.; 9 Department of Pathology, Yale University School of Medicine, New Haven, Connecticut, USA.; 10 Department of Biomedical Engineering and; 11 Wisconsin Blood Cancer Research Institute, University of Wisconsin–Madison, Madison, Wisconsin, USA.

**Keywords:** Clinical Research, Oncology, Cancer, Obstetrics/gynecology, Tumor suppressors

## Abstract

**BACKGROUND:**

A priori knowledge of recurrence risk in patients with nonmetastatic (International Federation of Gynecology and Obstetrics [FIGO] stage I) uterine serous carcinoma (USC) would enable a risk-stratified approach to the use of adjuvant chemotherapy. This would greatly reduce treatment-related morbidity and be predicted to improve survival.

**METHODS:**

GATA2 expression was scored by IHC across a retrospective multiinstitutional cohort of 195 primary USCs. Associations between GATA2 levels and clinicopathologic metrics were evaluated using Student’s *t* test, Fisher’s exact test, Kaplan-Meier method, and Cox proportional hazard ratio. Invasion in patient-derived USC cells was assessed by Student’s *t* test. RNA-Seq, anti-GATA2 ChIP-Seq, and confirmatory Western blotting enabled identification of GATA2 targets.

**RESULTS:**

Patients with FIGO stage I GATA2^hi^ USCs had 100% recurrence-free and 100% cancer-related survival, which was significantly better than patients with GATA2^lo^ USCs. In patients for whom adjuvant chemotherapy was omitted, patients with GATA2^hi^ USC had 100% recurrence-free 5-year survival compared with 60% recurrence-free survival in patients with GATA2^lo^ USC. Depletion of GATA2 in patient-derived USC cells increased invasion in vitro.

**CONCLUSION:**

Routine GATA2 IHC identifies 33% of patients with FIGO stage I USC who have a greatly reduced risk of posthysterectomy USC recurrence. Our results suggest that a GATA2-guided personalized medicine approach could be rapidly implemented in most hospital settings, would reduce treatment-related morbidity, and would likely improve outcomes in patients with USC.

**FUNDING:**

NIH grants R01 DK068634, P30 CA014520, S10 OD023526, K08 DK127244, T32 HL007899, the UW-Madison Department of Pathology and Laboratory Medicine, the UW-Madison Centennial Scholars Program, the Diane Lindstrom Foundation, the American Cancer Society, the V Foundation, The Hartwell Foundation, and the UMN Department of Obstetrics, Gynecology, and Women’s Health.

## Abstract 2

Loss of GATA2 expression leads to uterine serous carcinoma invasion and metastasis. Routine GATA2 immunohistochemistry predicts cancer recurrence and patient survival in FIGO stage I uterine serous carcinoma.

## Graphical abstract

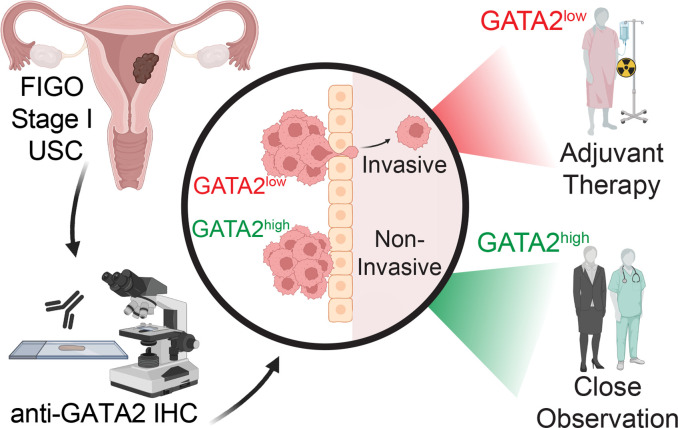

## Introduction

Uterine cancer is the fourth most common cancer in women, and its incidence is increasing (1). Regrettably, the survival rate for uterine cancer has been stagnant since the mid-1970s, and mortality rates have increased by 2% each year for the past decade (2). Worsening outcomes are primarily due to a rise in the incidence of high-risk uterine cancer subtypes, especially uterine serous carcinoma (USC). Whereas the yearly incidence of low-grade uterine cancers was stable between 2001 and 2017, the incidence of USC increased by 4.9% annually (3). USC arises in postmenopausal women, is highly aggressive, and carries a dismal 5-year cancer-related survival of 50% (4). Although USC constitutes only 10% of total uterine cancer cases, it is responsible for 40% of uterine cancer deaths (2).

USCs invade and metastasize rapidly, and 40% of patients with USC have metastatic disease at diagnosis due to spread through the uterine lymphatics or direct extension into the pelvic cavity (5). In contrast, 50% of patients with USC have nonmetastatic disease that is limited to the uterine body at diagnosis, a finding that corresponds to International Federation of Gynecology and Obstetrics (FIGO) stage I disease (Supplemental Table 1; supplemental material available online with this article; https://doi.org/10.1172/jci.insight.187073DS1). These patients should be cured by staging hysterectomy. However, USC will ultimately recur in 30% of these patients, an event that is associated with poor outcomes (6).

Contemporary molecular, surgical, histologic, and imaging modalities cannot predict which patients with FIGO stage I USC will recur after surgery. For this reason, the National Comprehensive Cancer Network (NCCN) guidelines recommend that physicians consider adjuvant chemotherapy with or without radiation in virtually all patients with FIGO stage I USC. This recommendation is made with the realization that adjuvant therapy benefits only a small subset of these patients in the form of reduced risk of USC recurrence (7, 8). However, the median age at USC diagnosis is 72 years and chemotherapy results in high rates of treatment-related morbidity in this age group, including neuropathy, low blood counts, fatigue, gastrointestinal side effects, and neurocognitive changes (9). If physicians can predict which patients with FIGO stage I USC will recur, adjuvant chemotherapy will improve survival in this group (8, 10), while patients with tumors predicted to be cured by hysterectomy could be spared unnecessary cytotoxic treatments, greatly reducing morbidity. This would represent a paradigm shift in USC treatment and would improve patient outcomes.

GATA binding protein 2 (GATA2) is a zinc finger transcription factor critical for the development and function of multiple organ systems (11). In the endometrium, GATA2 functions in an autoregulatory positive feedback loop with the progesterone receptor (PR) to regulate PR expression while also colocalizing with PR on chromatin to cooperatively contribute to PR transcriptional programs (12). Dysregulated GATA2 levels and activity are also implicated in the pathogenesis of both hematologic and solid malignancies (13, 14). Recently, *GATA2* RNA levels were reported to be reduced in endometrial carcinomas compared with benign endometrium in an analysis that did not stratify by tumor subtype (15). In that study, low *GATA2* RNA expression correlated with reduced survival time. Using cell lines derived from endometrioid (non-USC) endometrial carcinomas, the authors showed that increased GATA2 expression can suppress tumor growth and invasion (15).

Here, we utilized custom anti-GATA2 antibodies and performed GATA2 IHC on a multiinstitutional retrospective cohort of 195 total primary USCs (Supplemental Figure 1), including 127 FIGO stage I USCs. We show that routine GATA2 IHC identifies 33% of FIGO stage I USCs with high GATA2 expression (GATA2^hi^) and that patients with GATA2^hi^ USCs do not experience cancer recurrence after hysterectomy, even when adjuvant therapy is omitted.

## Results

### GATA2 IHC predicts recurrence and survival in USC.

We first performed GATA2 IHC on a retrospective cohort (UW1 cohort; Supplemental Table 2) of 70 primary USC tumors representing all FIGO stages that were removed at the time of staging hysterectomy, prior to any adjuvant therapy. This cohort was chosen because it had been previously assembled by one of the coauthors for prior studies (5, 16). All patients were diagnosed between 2003 and 2012, and patient follow-up extended through 2019. When compared using univariate and multivariate analyses to 1,710 patients in the SEER (https://seer.cancer.gov/) database diagnosed with USC from 2018 to 2021 and for whom sufficient clinicopathologic metrics were available for analysis, our UW1 cohort had similar patient age at diagnosis, tumor size, FIGO stage, rate of neoadjuvant chemotherapy, and rate of adjuvant chemotherapy (Supplemental Tables 3 and 4). However, our UW1 cohort had a significantly higher percentage of white women and significantly lower rates of adjuvant radiotherapy administration than those reported in SEER. The primary outcomes analyzed were: (a) recurrence free survival, defined as survival without recurrence of USC; (b) cancer-related survival, defined as no death due to USC; and (c) overall survival, defined as no death due to any cause.

**Figure 1:**
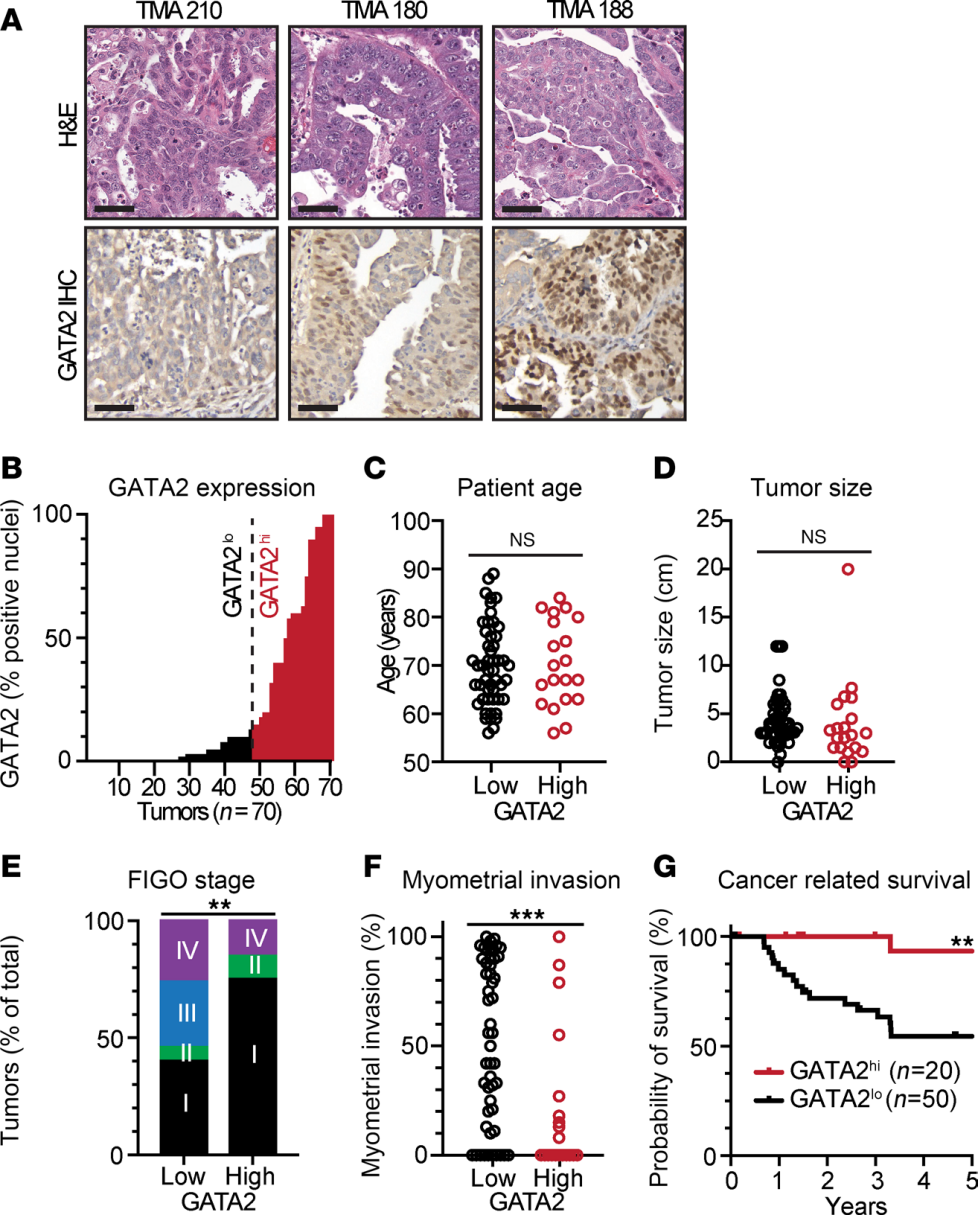
GATA2 IHC correlates with low FIGO stage and with cancer-related and overall survival in USC. (**A**) H&E sections and GATA2 IHC from representative USCs showing spectrum of GATA2 IHC staining. Tumor TMA 210 is an example of a GATA2^lo^ USC, while tumors TMA 180 and TMA 188 are both GATA2^hi^. H&E and DAB, 600×. Scale bar: 50 μm. (**B**) Histogram depicting percent GATA2^+^ tumor nuclei in all 70 cases from UW1 USC cohort. Vertical dotted line indicates 15% cutoff between GATA2^lo^ and GATA2^hi^ USCs. (**C**–**F**) Distribution of patient age (**C**), tumor size (**D**), FIGO stage (**E**), and myometrial invasion (**F**) between GATA2^lo^ and GATA2^hi^ USCs (*n* = 70). (**G**) Kaplan-Meier curve depicting cancer-related survival in USCs from UW1 cohort based on GATA2^lo^ or GATA2^hi^ status (*n* = 64). Analysis in **C**, **D**, and **F** by Student’s *t* test and analysis in **E** by Fisher’s exact test. **P < 0.005, ***P < 0.0005.

For GATA2 IHC, we utilized a mouse anti-GATA2 monoclonal antibody, which we previously demonstrated to be sensitive and specific for mouse and human GATA2 across diverse assays, including Western blot of whole cell lysate and IHC performed on benign and pathologic formalin-fixed paraffin-embedded endometrial biopsies (17, 18). To further confirm antibody specificity, we also performed IHC on the same cohort using a unique and extensively validated anti-GATA2 rabbit polyclonal antibody pool (19–22), which yielded an identical staining pattern as the monoclonal antibody (Supplemental Figure 2). We also previously performed anti-GATA2 immunoprecipitations (IPs) from whole cell lysate using the validated rabbit polyclonal anti-GATA2 antibody and showed that our anti-GATA2 monoclonal specifically recognized GATA2 in the anti-GATA2 IP lane relative to the species and isotype control lane (17). Tumor nuclei were either negative or positive for GATA2 (Figure 1A), which is a pattern of GATA2 expression that we have previously reported in benign and premalignant uterine tissues (18), as well as in myelodysplastic neoplasms and acute leukemias in the bone marrow (17). Because expression of related GATA family members GATA3 and GATA6 has been reported in uterine tissue (23, 24), we also performed GATA3 and GATA6 IHC on our UW1 cohort and confirmed that GATA3 and GATA6 expression did not correlate with GATA2 (Supplemental Figure 3).

The percentage of GATA2^+^ USC tumor nuclei ranged from 0% to 100% (Figure 1B), with an initial inflection point at 15% that we later found clearly separated patients by survival. In selecting a cutoff value for USC GATA2 scoring, we also sought to establish a straightforward GATA2 IHC scoring method amenable to routine surgical pathology practice. Therefore, we established 15% GATA2^+^ nuclei as a cutoff and assigned all USCs with fewer than or equal to 15% GATA2^+^ nuclei as GATA2^lo^, and USCs with greater than 15% GATA2^+^ tumor nuclei as GATA2^hi^ (Figure 1B).

Patients with GATA2^hi^ USCs were of similar age (Figure 1C) and had tumors that were the same size (Figure 1D) as patients with GATA2^lo^ USCs. However, patients with GATA2^hi^ USC presented at lower FIGO stage (Figure 1E) and showed significantly reduced myometrial invasion (Figure 1F), reduced uterine lymphovascular invasion (LVI), and were more frequently positive for estrogen receptor (ER) and PR compared with GATA2^lo^ USCs (Supplemental Table 5). Patients with GATA2^hi^ USC also had significantly better cancer-related and overall survival compared with patients with GATA2^lo^ USC (Figure 1G and Supplemental Figure 4A), and GATA2 status was independently predictive of overall survival (Supplemental Table 6). We also analyzed *GATA2* RNA transcript levels in 2 of the largest publicly available patients with USC cohorts (25, 26) and found no significant predictive value of *GATA2* transcript levels for survival, suggesting that RNA transcript levels are less informative than protein-based analyses (Supplemental Figure 4, B–E).

Interestingly, 75% of GATA2^hi^ USCs were from patients with cancer limited to the uterine body at diagnosis, representing FIGO stage I disease. Among the 30 patients in our UW1 cohort with metastatic, FIGO stage III–IV USC, only 3 (10%) had GATA2^hi^ USCs. Therefore, to further elucidate relationships between GATA2 expression and survival in patients with metastatic USC, we scored GATA2 IHC on an additional independent retrospective USC cohort (Yale cohort, *n* = 36; Supplemental Table 7), which included 28 primary tumors from patients with FIGO Stage III–IV USC (27). We analyzed the GATA2 status in the combined cohort of 58 metastatic USCs (FIGO stage III–IV tumors from the UW1 and Yale cohorts, *n* = 58; Supplemental Table 8). This revealed no significant relationship between GATA2 status and survival (Supplemental Figure 5). We concluded that GATA2 expression predicts recurrence and survival in USC but only in patients with nonmetastatic USC. Therefore, we focused further analyses on patients diagnosed with USC limited to the uterine body. These patients with FIGO stage I USC represent 50% of new USC diagnoses.

### GATA2 status predicts cancer recurrence and survival in FIGO stage I USC.

Under FIGO staging criteria (and similar American Joint Committee on Cancer criteria) (28), FIGO stage IA USCs are either noninvasive or invasive but penetrate less than 50% of the uterine myometrium, while FIGO stage IB USCs invade through 50% or more of the myometrium but remain contained within the uterine body. In both cases, there is no evidence of USC outside of the body of the uterus. It is recommended that surgical staging be performed in these patients, to include removal of the uterus, cervix, ovaries, fallopian tubes with lymph node evaluation (using sentinel lymph node removal or complete pelvic and para-aortic lymphadenectomy) and omental biopsy. This identifies microscopic disease and ensures accurate stage assignment.

Our initial UW1 USC cohort included 35 FIGO stage I USCs, and our Yale cohort included 3 FIGO stage I USCs. We augmented these cases by generating 2 new and unique retrospective cohorts representing 89 additional FIGO stage I USCs. The first new cohort consisted of 45 USC tumors derived from FIGO stage IA and IB patients who had been treated at the University of Wisconsin Hospital and Clinics (UW2 cohort, *n* = 45; Supplemental Table 9). GATA2 IHC and scoring was performed similarly to the UW1 cohort, except whole slides of tumor were stained and scored. For the second new cohort, the anti-GATA2 monoclonal antibody was independently optimized for IHC by the University of Minnesota (UMN) Medical Center (*n* = 44; Supplemental Table 10) where GATA2 IHC was scored blindly on 44 FIGO stage IA noninvasive USC tumors derived from UMN patients. Scoring at UMN was performed blindly by a senior UMN gynecologic pathologist and a pathology resident who were asked to score the percent GATA2^+^ tumor nuclei without any additional training. The total pooled FIGO stage I cohort consisted of patients pooled from the UW1, UW2, Yale, and UMN cohorts. This represented 127 total FIGO stage I USCs with matched survival data, a median follow-up period of 3.2 years, and variable amounts of matched clinicopathologic metrics (Table 1 and Supplemental Figure 1).

**Figure 2:**
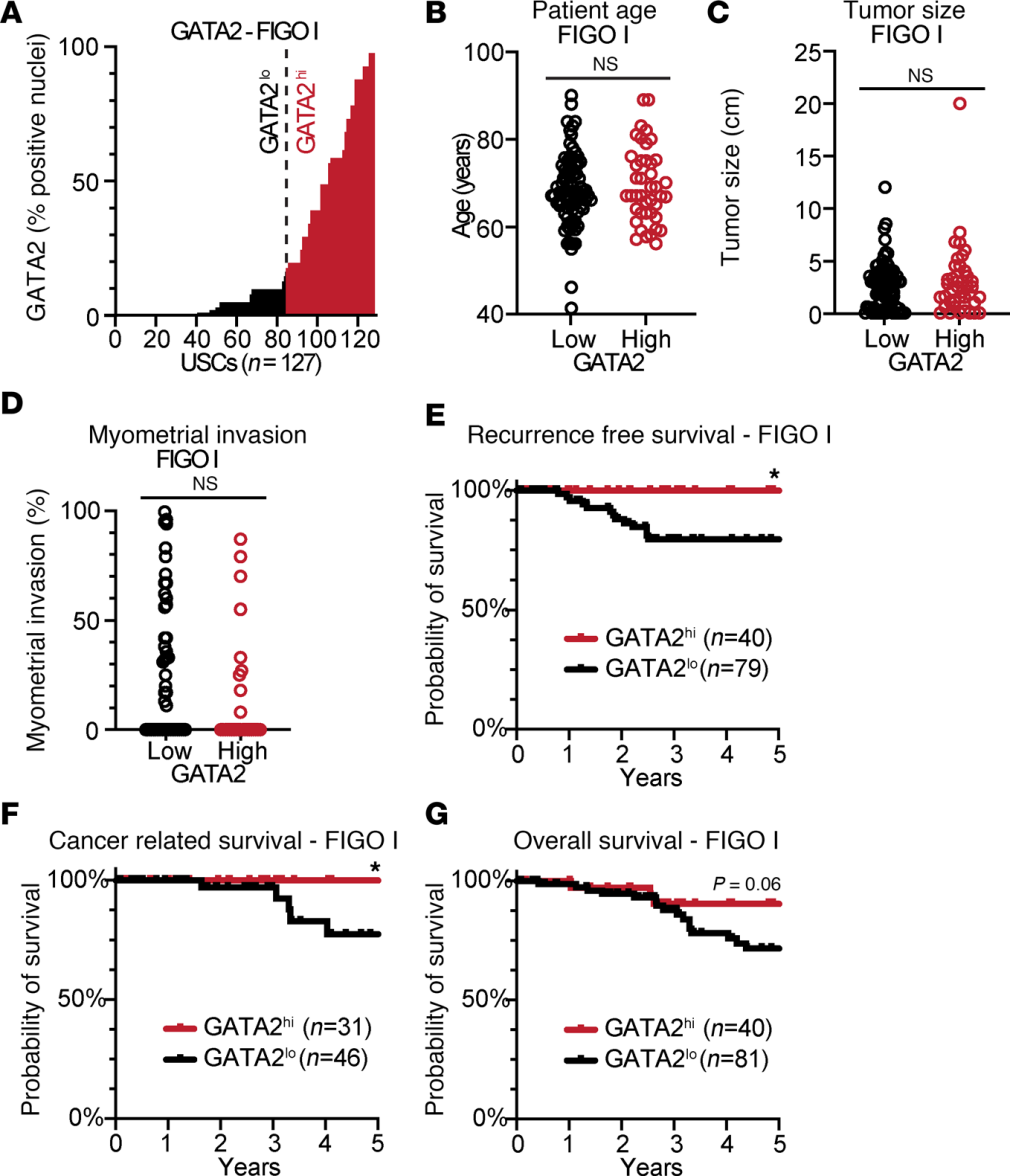
GATA2 IHC predicts recurrence and survival in FIGO stage I USC. (**A**) Histogram depicting percent GATA2^+^ tumor nuclei in combined FIGO stage I USC cohort (*n* = 127). These patients were pooled from UW1, UW2, Yale, and UMN cohorts. Vertical dotted line indicates 15% cutoff between GATA2^lo^ and GATA2^hi^ USCs. (**B**–**D**) Spectrum of patient age (*n* = 127) (**B**), tumor size at staging hysterectomy (*n* = 124) (**C**), and myometrial invasion (*n* = 121) (**D**) in FIGO stage I GATA2^lo^ and GATA2^hi^ USCs as analyzed by Student’s *t* test. (**E**–**G**) Kaplan-Meier curves depicting recurrence free (*n* = 123) (**E**), cancer-related (*n* = 78) (**F**), and overall survival (*n* = 121) (**G**) in FIGO stage I–II USCs based on GATA2^lo^ or GATA2^hi^ status. *P < 0.05.

We found that 33% of FIGO stage I USCs were GATA2^hi^ (Figure 2A). Patients with GATA2^hi^ FIGO stage I USCs were the same age at diagnosis (Figure 2B) and had the same BMI (Supplemental Table 11) as patients with FIGO stage I GATA2^lo^ USCs. In addition, GATA2^hi^ FIGO stage I USCs had the same tumor size (Figure 2C), the same incidence of uterine LVI and positive pelvic washings, and the same expression of ER, PR, and Erb-B2 Receptor Tyrosine Kinase 2 (Her2) compared with GATA2^lo^ USCs (Supplemental Table 11). GATA2^hi^ tumors also had the same degree of myometrial invasion as GATA2^lo^ USCs (Figure 2D). We attribute this to the fact that 67% of our FIGO stage I cohort is composed of FIGO stage IA noninvasive USCs, which by definition have no measurable myometrial invasion. Patients with GATA2^hi^ FIGO stage I USCs showed significantly better recurrence free (*P* = 0.01) and cancer-related 5-year survival (*P* = 0.04), and trended toward better overall survival (*P* = 0.06) compared with GATA2^lo^ USCs (Figure 2, E–G, and Supplemental Table 12). Notably, the 33% of FIGO stage I patients with GATA2^hi^ USCs showed 100% recurrence free and cancer-related survival.

### GATA2 depletion promotes invasion in patient-derived USC cells.

**Figure 3:**
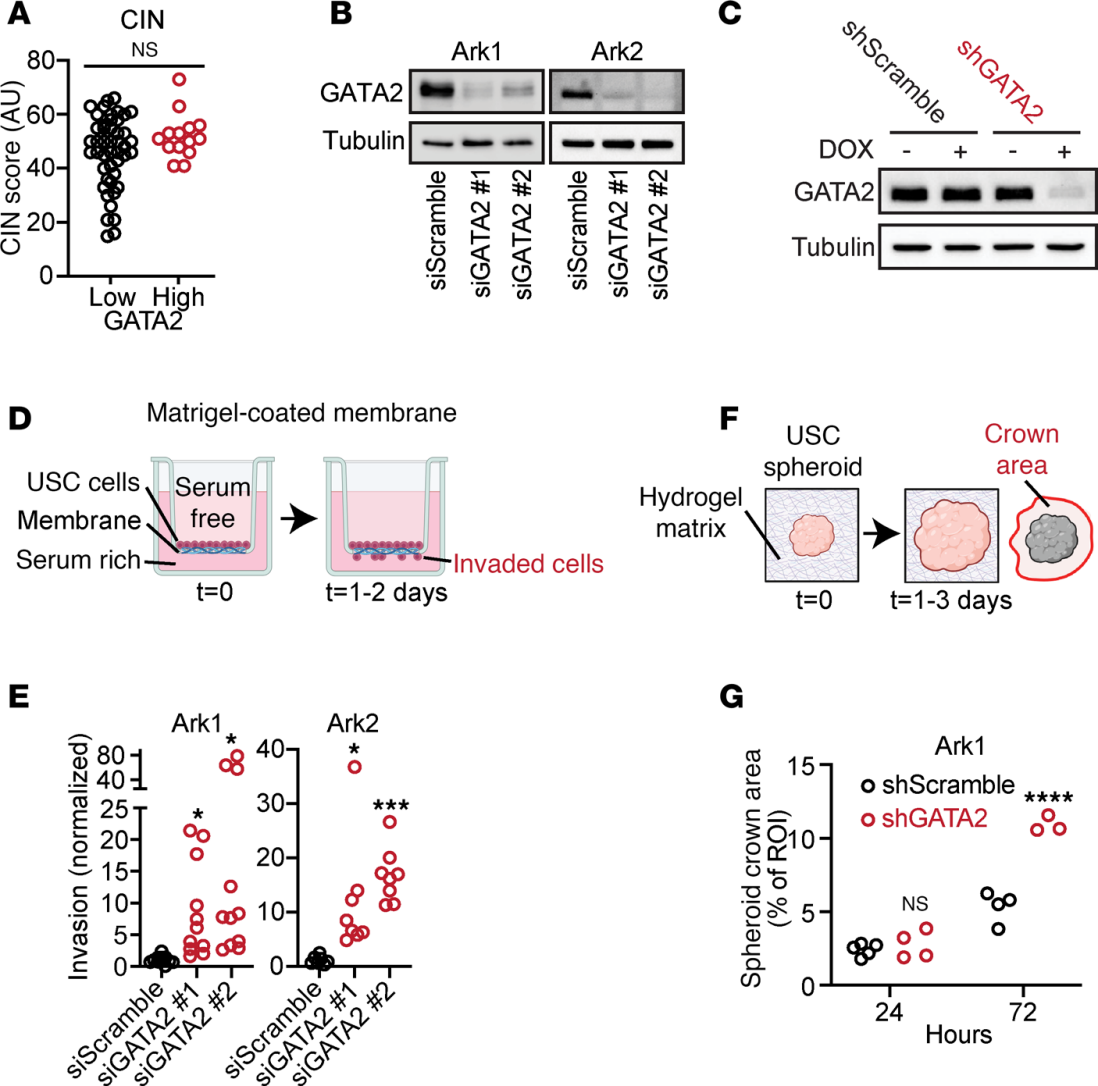
GATA2 depletion promotes invasion in patient-derived USC cell lines. (**A**) Level of chromosomal instability in GATA2^lo^ or GATA2^hi^ USCs (*n* = 60). (**B** and **C**) GATA2 is expressed in Ark1 and Ark2 patient-derived USC cells and can be depleted by anti-GATA2 siRNA (**B**) and doxycline-inducible shRNA (**C**). (**D**) Schematic of Matrigel-coated membrane invasion assay. (**E**) Ability of Ark1 and Ark2 USC cells to invade through Matrigel-coated membranes after GATA2 depletion (*n* = 11 for Ark1 and *n* = 7–8 for Ark2). (**F**) Schematic of hydrogel invasion assay. (**G**) Ability of Ark1 spheroids to invade into hydrogel after GATA2 depletion (*n* = 3–5). Analysis in **E** and **G** by Student’s *t* test, with Bonferroni corrections performed for both groups in **E**. *P < 0.05, ***P < 0.005, ****P < 0.0005. CIN, chromosomal instability; DOX, doxycycline.

We hypothesized that GATA2 expression suppressed USC invasion and metastatic spread, and we sought to test whether GATA2 plays a mechanistic role in USC biology or if GATA2 levels simply correlate with aggressive USC behavior. USC pathogenesis occurs in a stepwise manner, in which foundational inactivation of TP53 is followed by acquisition of chromosomal instability (CIN), leading to a “copy number high” state that promotes USC invasion and metastasis through unclear mechanisms (25). Since 93% of USCs in our UW1 cohort were TP53 inactivated (Supplemental Table 2) despite tumors having GATA2 expression ranging from 0% to 100%, it indicated that GATA2 loss did not occur prior to foundational TP53 inactivation. To determine whether GATA2 loss occurs before or after acquisition of CIN, we performed CIN FISH on our UW1 USC cohort and found no significant difference in CIN between GATA2^hi^ and GATA2^lo^ USCs (Figure 3A), supporting a model whereby GATA2 loss occurs after TP53 inactivation and acquisition of CIN, and therefore represents a more proximal molecular readout of USC invasion and metastatic potential.

Using previously published Ark1 and Ark2 patient-derived USC cell lines (29), we next demonstrated that USC cells express GATA2, which we could deplete in a transient fashion using 2 unique pools of anti-GATA2 siRNA (Figure 3B and Supplemental Figure 6A) or long-term via a doxycycline-inducible anti-GATA2 shRNA (Figure 3C and Supplemental Figure 6B). Since there are 6 GATA factor family members with varying degrees of primary sequence homology and functional overlap (30), we quantified the expression of GATA family members in Ark1 and Ark2 cells using quantitative PCR (qPCR) after GATA2 depletion or scramble control (Supplemental Figure 7A). We detected *GATA6* expression in both Ark1 and Ark2 cells and detected *GATA3* only in Ark2 cells, neither of which showed significant changes in transcript levels after GATA2 siRNA treatment.

We then tested the effect of GATA2 depletion on USC invasion activity using 2 independent assays. First, we tested the ability of USC cells treated with siGATA2 to traverse Matrigel-covered membranes compared with siScramble controls (Figure 3D). In both Ark1 and Ark2 cells, siGATA2 treatment resulted in a significant increase in invasion compared with siScramble control (Figure 3E). We then sought to complement these findings in a more physiologically relevant system. To this end, we generated 3-dimensional tumor spheroids from Ark1 cells with doxycycline-inducible expression of shGATA2 or shScramble control. Inducible shRNAs were used, as lipofection required for siRNA delivery could not penetrate into tumor spheroids. After addition of doxycycline, spheroids were embedded in natural hydrogel casted within a microfluidic chamber. Over time, tumor invasion was quantified as increased crown area, defined as the invasive front of tumor cells escaping the spheroid core (Figure 3F). Over 72 hours, shGATA2 Ark1 cells showed significantly increased invasion compared with shScramble controls (Figure 3G).

Increased USC invasion was not solely due to increased USC proliferation. While GATA2 depletion did result in increased proliferation in Ark1 USC cells, there was no effect of GATA2 depletion on proliferation in Ark2 USC cells compared with controls (Supplemental Figure 7B). More importantly, GATA2^lo^ patient USC tumors were the same size as their GATA2^hi^ counterparts in patients with USC (Figure 1D and Figure 2C).

### GATA2 does not affect USC response to chemotherapy.

GATA2 expression has no effect on survival in patients with metastatic (FIGO stages III–IV) USC (Supplemental Figure 5). Because the survival of these patients is largely dependent on response to combination paclitaxel + carboplatin chemotherapy, it implies that GATA2 expression does not affect USC chemotherapy response. However, GATA2 IHC was performed on primary (uterus localized) tumors in our initial analysis, and following staging hysterectomy, it is the response of metastatic USC tumors to chemotherapy that determines patient survival. Therefore, we performed GATA2 IHC on metastatic tumors collected at staging hysterectomy from 22 patients in our UW1 cohort and compared their expression to the primary tumor to determine if USC metastases have different GATA2 levels than the primary tumors (Supplemental Figure 8). Interestingly, the average GATA2 expression in metastatic tumors was equal to the primary tumor in 8 cases (36%), less than the primary tumor in 9 cases (41%), and greater than the primary tumor in 5 cases (23%) (Supplemental Figure 8A). GATA2 expression varied even in different metastatic tumors from the same patient collected at different anatomic locations (Supplemental Figure 8B). Overall, in 77% of cases, metastatic USC tumors have equal to or less GATA2 expression than the primary tumor, but there is substantial variability in GATA2 expression in USC metastases. To test if altered GATA2 expression affected sensitivity to chemotherapy, we depleted GATA2 in Ark1 and Ark2 USC cells, treated them with paclitaxel, carboplatin, or combination paclitaxel + carboplatin, and analyzed cell survival after 96 hours compared with scramble controls (Supplemental Figure 9). This confirmed that GATA2 levels have no effect on USC sensitivity to chemotherapy, in line with USC survival data in FIGO stage III–IV patients (Supplemental Figure 5).

### GATA2 multi-omics identifies SIN3B as a USC GATA2 target gene.

**Figure 4:**
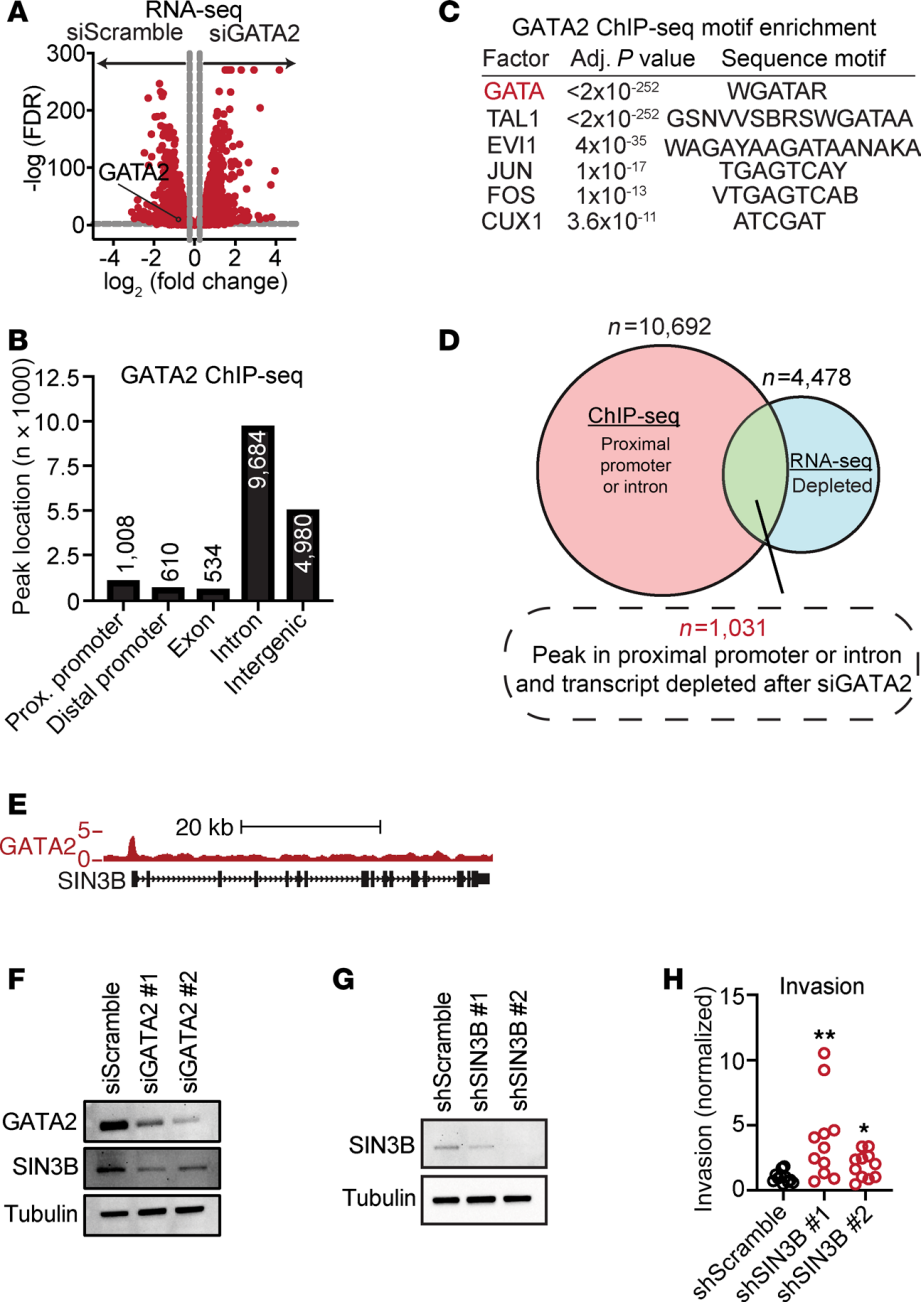
GATA2 multi-omics identifies SIN3B as a GATA2 target that regulates USC invasion. (**A**) Volcano plot of RNA-Seq data showing RNA expression changes after GATA2 depletion in Ark1 USC cells. (**B**) Number of genome wide GATA2 ChIP-Seq peaks that fall within different gene regulatory regions. (**C**) Transcription factor motifs enriched within regions of GATA2 ChIP-Seq occupancy. (**D**) Approach to identifying candidate GATA2-regulated genes in USC. (**E**) SIN3B locus with superimposed GATA2 ChIP-Seq occupancy. (**F**) Western blot depicting SIN3B levels after GATA2 depletion in Ark1 USC cells. (**G**) Western blot showing SIN3B levels after induction of shSIN3B or shScramble control in Ark1 USC cells. (**H**) USC invasion through Matrigel-coated membranes following depletion of SIN3B or scramble control (*n* = 11) as analyzed by Student’s *t* test with Bonferroni correction. *P < 0.05, **P < 0.005.

Since GATA2 is a transcription factor that functions through binding to genomic GATA nucleotide motifs and activating transcription of nearby target genes, we next sought to identify GATA2 networks and gene targets that may normally suppress USC invasion. To this end, RNA-Seq of Ark1 cells performed after GATA2 depletion revealed 8,500 differentially expressed genes (DEGs) (Figure 4A), and gene ontology (GO) term analysis of the top 500 most significantly upregulated and top 500 most significantly downregulated DEGs revealed downregulation of basement membrane reorganization and upregulation of protein localization to the cell cortex, integrin-mediated signaling pathways, and muscle cell migration, respectively (Supplemental Figure 10). We complemented RNA-level analyses by performing anti-GATA2 ChIP-Seq also in Ark1 cells, which revealed 16,816 genomic peaks of GATA2 occupancy, 64% (10,692 peaks) of which were in either proximal gene promoters or introns (Figure 4B). As expected, peaks of GATA2 occupancy were most enriched in GATA consensus nucleotide motifs but also showed significant enrichment for consensus binding motifs corresponding to TAL BHLH Transcription Factor 1 (TAL1), MDS1 and EVI1 Complex Locus (MECOM), Fos Proto-Oncogene (FOS), Jun Proto-Oncogene (JUN), and Cut Like Homeobox 1 (CUX1) (Figure 4C).

To identify putative direct GATA2 gene targets, we cross-referenced the genes in which GATA2 bound proximal promoters and/or introns with the genes whose expression was significantly reduced after GATA2 depletion, which yielded 1,031 genes (Figure 4D). To these, we applied a prioritization scheme that gave greater weight to genes based on degree of reduced expression after GATA2 depletion, size of the associated GATA2 occupancy peak, and published involvement in cancer progression. From this emerged SIN3 transcription regulator family member B (*SIN3B*), which codes for a conserved scaffold protein and histone deacetylase (HDAC) complex subunit that tethers HDAC activity to sequence-specific transcriptional repressors while also stimulating HDAC activity (31, 32). Reduced SIN3B levels impair cellular differentiation and cell cycle withdrawal in response to oncogenic stimuli (33, 34), and SIN3B is reported to variably promote or suppress tumor invasion depending on cancer type (35–37).

GATA2 occupies the *SIN3B* proximal promoter in USC cells (Figure 4E), and we confirmed that SIN3B levels are reduced after GATA2 depletion (Figure 4F), supporting a model whereby GATA2 normally promotes *SIN3B* expression. To test whether SIN3B normally suppresses USC invasion, similarly to GATA2, we generated doxycycline-inducible shSIN3B Ark1 cells (Figure 4G) and found that SIN3B-depleted Ark1 cells had a significantly increased capacity to invade through Matrigel-coated membranes compared with shScramble controls (Figure 4H). Finally, we performed IHC for SIN3B on our UW1 cohort and scored both the percent SIN3B^+^ tumor nuclei and the intensity of SIN3B staining. However, we found no significant relationship between SIN3B and GATA2 expression (Supplemental Figure 11), indicating that GATA2 regulation of SIN3B may not be generalizable across all USC tumor contexts.

### GATA2 IHC predicts FIGO stage I USC recurrence in the absence of adjuvant therapy.

**Figure 5:**
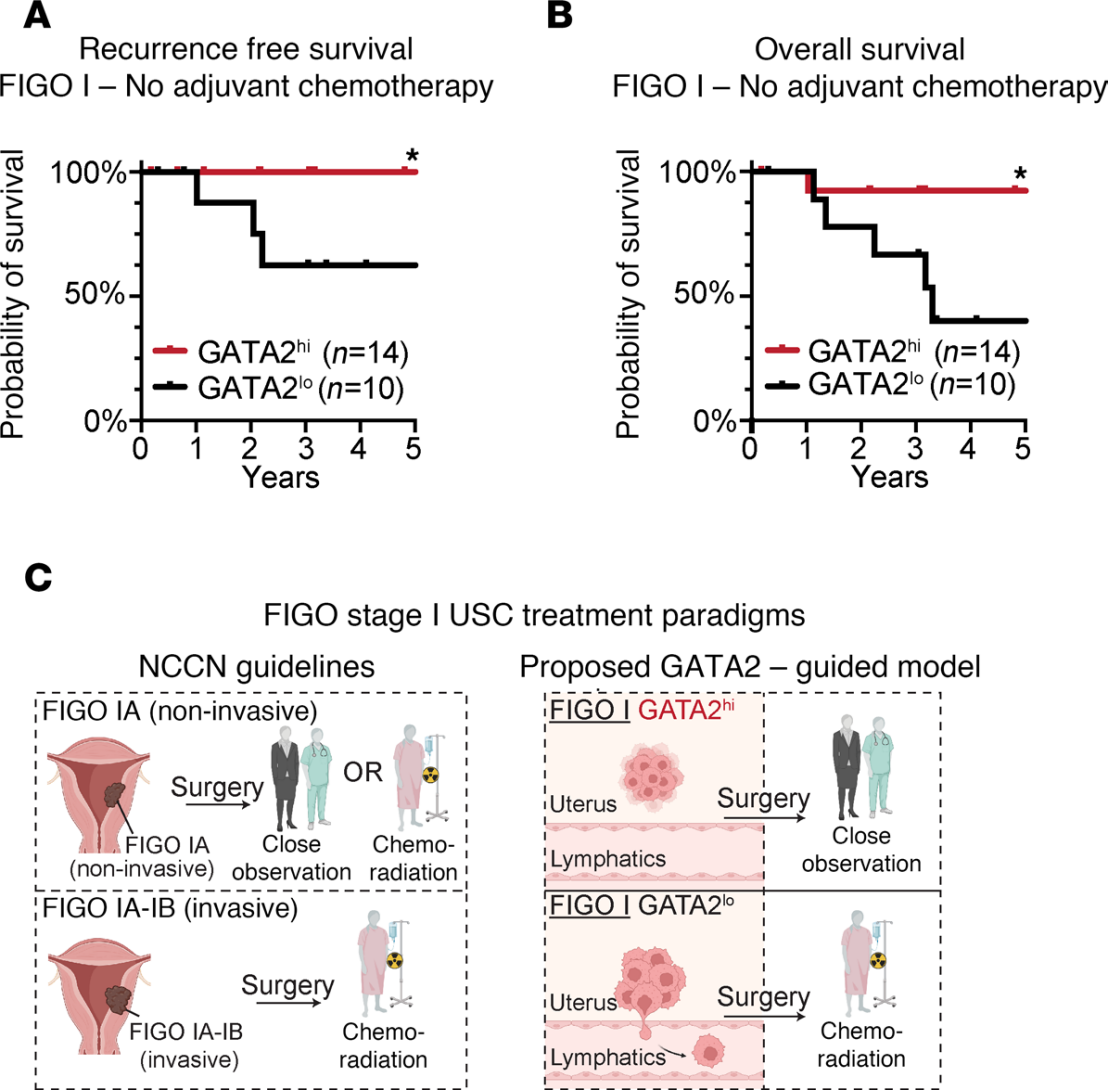
GATA2 IHC predicts recurrence and overall survival in FIGO stage I USC in the absence of adjuvant chemotherapy. (**A** and **B**) Kaplan-Meier curves depicting recurrence-free (*n* = 24) (**A**) and overall survival (*n* = 24) (**B**) in GATA2^lo^ and GATA2^hi^ patients with FIGO stage I USC who did not receive adjuvant chemotherapy. These patients were pooled from UW1, UW2, Yale, and UMN cohorts. (**C**) Current NCCN guidelines and proposed GATA2-guided treatment model for FIGO stage I USC. Per the NCCN, patients with FIGO stage IA noninvasive USCs may receive either close observation or adjuvant therapy, whereas patients with invasive FIGO stage I USCs should receive adjuvant therapy. In a proposed GATA2-guided model, patients with GATA2^hi^ FIGO stage I USCs would be offered close observation, while patients with GATA2^lo^ FIGO stage I USC would be offered adjuvant therapy. *P < 0.05. NCCN, National Comprehensive Cancer Network.

If GATA2^hi^ FIGO stage I USCs have a greatly reduced capacity to invade and metastasize, and therefore have very high rates of cure by staging hysterectomy, then adjuvant therapy is unlikely to be critical in reducing the rate of USC recurrence in these patients. Moreover, we showed that GATA2 expression does not affect chemotherapy response, again suggesting that adjuvant therapy should play no role in a GATA2-related survival benefit. If GATA2 IHC could predict USC recurrence, then 33% of patients with FIGO stage I USC with GATA2^hi^ tumors could be spared adjuvant therapy and instead undergo a period of close clinical observation. To evaluate this possibility, we identified 24 patients in our combined cohort who had FIGO stage I USC and received no adjuvant or neoadjuvant chemotherapy, including 14 GATA2^hi^ USCs and 10 GATA2^lo^ USCs (*n* = 24; Supplemental Table 13). These patients represented all patients with FIGO stage I USC from our UW1, UW2, Yale, and UMN cohorts who had not received adjuvant chemotherapy. Among these chemotherapy naive patients, those with GATA2^hi^ USCs had 100% recurrence free survival, which was significantly better than patients with GATA2^lo^ USCs (Figure 5A). The same patients with GATA2^hi^ USCs also had significantly improved overall survival compared with patients with GATA2^lo^ USCs (Figure 5B).

## Discussion

NCCN guidelines (version 2.2024) recommend that most patients with FIGO stage I USC receive adjuvant chemotherapy with carboplatin and paclitaxel ± radiation (7). In FIGO stage IA noninvasive USC with negative pelvic washings, patients may elect to receive only local vaginal brachytherapy with the option of chemotherapy. Alternatively, these patients may receive no adjuvant therapy and choose only clinical observation. In our cohort of patients with FIGO stage I USC, 79% of women opted for adjuvant chemotherapy and 72% received adjuvant radiotherapy. The NCCN guidelines recommend that only patients with no identifiable residual tumor at surgical staging forego adjuvant treatment and receive only observation, but these patients are very uncommon and represented only 4% of our USC FIGO stage I cohort.

Clinicians are aware that the current treatment paradigm results in many patients with USC receiving adjuvant chemoradiation who were likely cured by hysterectomy. Clinicians also recognize the considerable morbidity and rarely mortality associated with chemoradiation therapy in this patient population, which has a median age of 72 years. However, up to 30% of patients with FIGO stage I USC will experience diseased recurrence, and there are no reliable markers to predict which patients will experience recurrence. Because adjuvant chemotherapy reduces the risk of USC recurrence (38), many patients with USC are treated so that a minority of patients with USC whose cancer will recur can experience a recurrence and survival benefit (Figure 5C). We believe that precision medicine in the form of routine GATA2 IHC performed at diagnosis could fundamentally upend this treatment paradigm and guide adjuvant therapy toward patients at high risk of USC recurrence while sparing large numbers of very low–risk patients from chemotherapy and radiation (Figure 5C).

Our study has some important caveats. First, USC is a relatively uncommon malignancy, and we have identified few available large tissue cohorts with linked detailed clinicopathologic data. Our multiinstitutional case cohort includes 195 total USC cases, including 127 FIGO stage I cases. While mostly representative of population-wide patients with USC, White women were overrepresented in our cohort. Given the marked racial disparities in USC outcome, we intend to assemble a much larger and racially representative USC cohort in collaboration with additional extrainstitutional partners to confirm the utility of GATA2 IHC across the full USC patient demographic. Second, our data were acquired retrospectively and our study cannot substitute for a carefully controlled prospective clinical trial, which is in the planning phase. Third, 70% of GATA2 IHC from primary USCs was performed on tissue microarrays, whereby small portions of tumors are arrayed as circular punches on a single slide. In addition, while GATA2 IHC was crisp and nuclear in all cases, less than 5% of cases also showed faint cytoplasmic staining. Cytoplasmic staining was interpreted as off-target labeling due to the absence of a known cytoplasmic pool of GATA2, and cytoplasmic staining was not predictive of USC behavior or patient outcome. It will be critical to ensure that GATA2 IHC can be easily performed and scored on full sections of tumor such as those evaluated in routine surgical pathology practice, and that IHC labeling is uniform across additional institutions and centers. Finally, the full molecular mechanisms driving USC invasion and metastasis after GATA2 loss remain to be elucidated. Our identification of SIN3B as a putative GATA2 USC target gene serves as a model for how such factors can be identified using rigorous multi-omic approaches. However, as we found that SIN3B protein levels measured by IHC did not clearly correlate with GATA2 across a larger USC tumor cohort, it is likely that clinically relevant GATA2 regulation of SIN3B may not extend across the full USC tumor spectrum. As GATA2 regulation is often highly context dependent, this is not surprising, and it supports deeper investigations of GATA2 USC mechanisms with the goal of identifying the most critical GATA2-regulated drivers of USC invasion and metastasis. These new insights into GATA2 biology will inform any future application of GATA2 IHC.

Our findings raise the possibility that routine GATA2 IHC may provide clinicians with a powerful tool as they seek to balance treatment goals with quality of life for patients with USC. Establishing this will require carefully structured prospective studies. Our validated anti-GATA2 monoclonal antibody has already been optimized for standard surgical pathology workflows (17), ensuring that GATA2 IHC could be rapidly implemented in future USC treatment paradigms at any major medical center.

## Methods

### Sex as a biological variable.

USC only occurs in women; therefore, all patients in this study are women, and all patient-derived USC cell lines are from women.

### USC tissue microarrays and IHC.

Construction and case composition of both tissue microarrays were reported earlier (16, 27). IHC was performed on an automated Ventana Discovery Ultra BioMarker platform. IHC protocols for custom rabbit anti-GATA2 polyclonal antibody and mouse custom anti-GATA2 15D2 monoclonal antibody also have both been reported (17, 18) and show essentially identical IHC staining patterns (Supplemental Figure 2). GATA6 IHC was performed using a commercial antibody (Cell Signaling Technology, 5851). Briefly, deparaffinization was carried out on the instrument, as was heat-induced epitope retrieval with cell conditioner 1 buffer (Ventana, 950-224) for 32 minutes at 95°C. GATA6 antibody was diluted 1:750 with DaVince green diluent (Biocare Medical, PD900H). Slides were incubated with primary antibody for 32 minutes at 37°C before being rinsed with reaction buffer (Ventana, 950-300), incubated with Discovery OmniMap anti–rabbit horseradish peroxidase (Ventana, 760-4311) for 16 minutes at 37°C, and rinsed with reaction buffer. Discovery ChromoMap DAB detection kit (Ventana, 760-159) was used for visualization. The slide was removed from the instrument and rinsed, counterstained with Harris hematoxylin (1:5) for 45 seconds, rinsed again, dehydrated in an oven, and dipped in xylene before addition of mounting media and a coverslip. GATA3 IHC was performed using a commercial antibody (Ventana, clone L50-823) under similar conditions as the GATA6 IHC, except epitope retrieval was performed for 16 minutes and incubation in GATA3 antibody for 12 minutes. SIN3B IHC was performed using a commercial antibody (Novus, NBP2-20367) in a manner similar to GATA6 IHC with the following exceptions: epitope retrieval with CC2 buffer (Ventana, 760-107) for 64 minutes, primary antibody incubation for 12 hours at room temperature, and visualized with Discovery OmniMap anti-Rabbit HRP (Ventana, 760-4311) for 16 minutes at 37°C.

IHC scoring of the UW1 and UW2 cohorts was performed by a board-certified anatomic pathologist using an Olympus BX41 widefield microscope. All scoring was performed in a blinded fashion, with pathologists having no prior knowledge of clinicopathologic metrics or patient outcome. Individual IHC scores for each case represented an average taken across all available tissue cores for each case. All images were white balanced in Adobe Photoshop for publication.

### CIN FISH and scoring.

CIN FISH was performed as reported previously (39). FISH enumeration of chromosomes 3, 4, 7, 9, 10, and 17 was completed using the following 2 probe mixes: Vysis CEP 3 (D3Z1) labeled SpectrumOrange (Vysis,06J3613) localizing to 3p11.1–q11.1, Vysis CEP 7 (D7Z1) labeled SpectrumAqua (Vysis, 06J5427) localizing to 7p11.1–q11.1, Vysis CEP 9 labeled SpectrumGreen (Vysis, 06J3719) localizing to 9p11–q11 in IntelliFISH hybridization buffer (Vysis, 08N8701), Vysis CEP 4 labeled SpectrumGreen (Vysis, 06J3714) localizing to 4p11–q11, Vysis CEP 10 labeled SpectrumAqua (Vysis, 06J5420) localizing to 10p11.1–q11.1, and Vysis CEP 17 (D17Z1) labeled SpectrumOrange (Vysis, 06J3697) localizing to 17p11.1–q11.1 in IntelliFISH hybridization buffer (Abbott Molecular). Chromosomes were counted by observers blinded to patient conditions in a minimum of 10 cells per case. Ploidy was determined by the average chromosome number counted on 6 probes combined. CIN was determined as the average percentage of cells that deviated from the modal number for each of the 6 chromosomes assessed by FISH.

### Cell culture, siRNA, shRNA, and proliferation assays.

Ark1 and Ark2 patient-derived ESC cells were purchased from Alessandro Santin (Yale University, New Haven, Connecticut, USA) with their derivation reported earlier (29). Cells were cultured in RPMI-1640 media (MilliporeSigma) with 10% FBS and 1% penicillin/streptomycin. Sequences for siRNAs are GATA2 pool #1 (5′-GCACAAUGUUAACAGGCCA-3′, 5′-GCGCACAACUACAUGGAAC-3′), GATA2 pool #2 (5′-GCUUCGAGGAGCUGUCAAA-3′, 5′-CCAACAAGUCCAAGAAGAG-3′), and Scramble pool (5′-UAGCGACUAAACACAUCAA-3′, 5′-UAAGGCUAUGAAGAGAUAC-3′, 5′-AUGUAUUGGCCUGUAUUAG-3′, 5′-AUGAACGUGAAUUGCUCAA-3′). Depletions were performed using Lipofectamine RNAiMax (Invitrogen) using 20 nM siRNA according to the manufacturer’s protocol, and depletion efficiency was measured after 48 hours by Western blotting using the same rabbit anti-GATA2 polyclonal antibody utilized for IHC. For shRNAs, stable doxycycline-inducible Ark1 or Ark2 lines were generated by transfecting cells with virions packaged in a pTRIPZ backbone (Horizon Discovery). After transfection, pools were selected for by treatment with 1 μg/mL puromycin, and shRNA induction with 1 μg/mL doxycycline was confirmed by IRES-dependent RFP expression; GATA2 depletion was confirmed by Western blot. shRNA-Seq were shGATA2 #1 (5′-TTCTCTACATAAAGTTGTC-3′) and shGATA2 #2 (5′-TCTTGCTCTTCTTGGACTT-3′).

For proliferation assays, cells were plated into 96-well plates and treated with siRNA the following morning. At 24 and 48 hours after siRNA treatment, cells were trypsinized and cell numbers were manually quantified with a hemocytometer. For invasion assays, cells were transferred to Matrigel-coated membrane inserts (membrane 8.0 μm) (Thermo Fisher Scientific, 8774122) 24 hours after siRNA treatment and allowed to migrate for an additional 24 hours, after which Matrigel was removed and the bottom surface of inserts was stained with Diff-Quick reagent and imaged by standard microscopy. Cell number was then quantified in a blinded fashion across 5 random fields.

### Spheroids and invasion assays.

For Matrigel invasion assays, cells were transferred to Matrigel-coated membrane inserts (membrane 8.0 μm) (Thermo Fisher Scientific, 8774122) 24 hours after siRNA treatment and allowed to migrate for an additional 24 hours, after which Matrigel was removed and the bottom surface of inserts was stained with Diff-Quick reagent and imaged by standard microscopy. Cell number was then quantified in a blinded fashion across 5 random fields.

Spheroids (2,500 cells/spheroid) were generated using the hanging drop method for Ark1 shGATA2 and shScramble control. Briefly, cells were suspended in relevant media with 20% methylcellulose suspension (12 g/L methylcellulose dissolved in RPMI serum-free media). In total, 25 μL droplets of cell suspension were placed on top of a Petri dish lid, and distilled water was added to the bottom of the dish to reduce evaporation during spheroid formation. After 2 days, a spheroid had formed in each droplet and was collected and utilized for subsequent hydrogel invasion assays.

Microdevices were fabricated using standard soft lithography as previously described (40–42). Briefly, polydimethylsiloxane-based (PDMS-based) microdevices were fabricated using the SU-8 template and bonded to a 60 mm glass bottom Petri dish (Mattek) using oxygen plasma followed by UV sterilization for 15 minutes. Final microdevices were composed of a central microchamber to inject a 3D hydrogel and a 280 μm–diameter PDMS rod placed in 1 of the side designated channels. To increase hydrogel attachment to the PDMS, microdevices were treated for 10 minutes with poly(ethyleneimine) (Sigma-Aldrich) diluted at 1%, followed by 30 minutes with glutaraldehyde (Sigma-Aldrich) diluted at 0.1% in water, and finally washed 4 times with water.

A collagen-spheroid suspension was prepared using rat tail type I collagen (Corning, 105.82 μL at 9.45 mg/mL), NaOH 1N to neutralize (MilliporeSigma, 9.4 μL), PBS 10X (Invitrogen, 30 μL), fibronectin (MilliporeSigma, 40 μL), and Matrigel (Corning, 60 μL) (43). Collected spheroids were then added into the collagen mixture (30 spheroids in 50 μL) and injected into the device chamber. Collagen hydrogels were polymerized upside down for 15 minutes at room temperature prior to transferring to a standard incubator for 30 minutes. Then, PDMS rods were removed using tweezers, and the resulting structure was filled (1.5 μL) with a Human Lymphatic endothelial cell suspension (ScienCell, passages 3–5) at 30,000 cells/μL in EGM-2 MV media (Lonza). Cells were incubated on both sides of the device for a total of 1 hour, and excess cells were washed out. In total, 4 mL of media + 1 μg/mL doxycycline was added to the plates and devices were imaged (10×, Leica Thunder *z* stacks in phase contrast and FITC channel, steps of 20 μm) after 24 and 72 hours. Stacks were *z*-projected using ImageJ (NIH), images were binarized, and consistent thresholds across conditions were used to quantify the area occupied by spheroid (core) and cell invaded area (crown), as the percentage of standard field of view.

### PCR.

Total RNA was purified from 1 × 10^5^ to 5 × 10^5^ cells with TRIzol (Invitrogen), and 2 μg RNA was treated with DNase I (Thermo Fisher Scientific) for 15 minutes at room temperature. After heat inactivation of DNase I with EDTA for 10 minutes at 65°C, RNA was incubated with 250 ng of a 4:1 mixture of oligo(dT) primers and random hexamer at 68°C for 10 minutes. RNA/primers were incubated with Moloney murine leukemia virus reverse transcription (M-MLV RT) (Thermo Fisher Scientific), 5× first strand buffer (Thermo Fisher Scientific), 10 mM dithiothreitol (Thermo Fisher Scientific), RNasin (Promega), and 0.5 mM deoxynucleoside triphosphates (New England Biolabs) at 42°C for 1 hour and then heat-inactivated at 95°C for 5 minutes. Quantitative gene expression analyses were performed using Power SYBR Green Master Mix (Applied Biosystems) and analyzed on a ViiA 7 Real-Time PCR System (Applied Biosystems). Control reactions without M-MLV RT yielded little to no signal. Relative expression of mRNA was determined from a standard curve of serial dilutions of cDNA samples, and values were normalized to 18S RNA expression. See Supplemental Table 14 for primers.

### Chemotherapy treatment assays.

Cells were plated into optical bottom 96-well plates and treated with siRNA the next morning. After 6 hours, the media were replaced with media containing drug or vehicle control. For combination carboplatin + paclitaxel treatment, cells received a constant 2.1 μM carboplatin and varying doses of paclitaxel. After 72 hours of incubation, cells were fixed and stained with DAPI before being imaged on a PerkinElmer Operetta high-content imaging system. The number of viable cells was scored based on DAPI staining intensity and distribution, following the application of filters trained to segregate out apoptotic debris.

### RNA-Seq library preparation, sequencing, and GO term analysis.

RNA-Seq studies were performed in triplicate using Ark1 cells, and comparisons were performed between treatment with Scramble siRNA versus GATA2 Pool #2 siRNA after 48 hours of siRNA treatment. RNA-Seq library preparation and sequencing were performed by the University of Wisconsin Biotechnology Center – Gene Expression Center. Briefly, sample purity and integrity were first analyzed on a NanoDrop One Spectrophotometer (Thermo Fisher Scientific) and 4200Tapestation (Agilent). Then 1 µg total RNA was enriched for mRNA by oligo(dT) selection followed by fragmentation using divalent cations under elevated temperature. Double-stranded cDNA was synthesized using SuperScript II RT (Invitrogen) and random primers for first-strand synthesis. Double-stranded cDNA was purified with SPRI beads (Beckman Coulter Genomics) followed by incubation with Klenow DNA polymerase and adenine for single base addition at blunt end termini. DNA fragments were ligated to Illumina unique molecular index adapters, purified with SPRI beads, and PCR amplified with a Phusion DNA polymerase and Illumina genomic DNA primer set, followed by SPRI bead purification. The quality and quantity of finished libraries were assayed by 4200 TapeStation using D1000 Screentape and Qubit HS Quantification Kit (Invitrogen). Paired-end 150 bp sequencing was carried out on an Illumina NovaSeq6000, with libraries multiplexed for approximately 30 million reads per library. GO term analysis was performed on the top 500 most overexpressed and top 500 most underexpressed transcripts as determined by FDR, using PANTHER (https://pantherdb.org/).

### Anti-GATA2 ChIP-Seq.

In total, 150 million Ark1 cells were incubated in serum free media with 1% paraformaldehyde for 10 minutes at room temperature, before being quenched with 125 mM glycine. Cells were scraped from the plate, pelleted for 5 minutes at 700*g*, and then resuspended in 9 mL of Buffer A (50 mM HEPES [pH 7.9] [DOT Scientific], 140 mM NaCl [DOT Scientific], 1 mM EDTA [MilliporeSigma], 10% glycerol [DOT Scientific], 0.5% NP-40 [US Biological], 0.25% Triton X-100 [Acros], and 1 tablet Pierce protease inhibitor cocktail mini) and incubated on ice for 10 minutes. The cells were then pelleted for 5 minutes at 1,400*g*, the supernatant was aspirated, and the pellet was resuspended in 3 mL Buffer A. After incubation on ice for 5 minutes, cells were pelleted for 5 minutes at 1,400*g*, and the supernatant was aspirated. The pellet was resuspended in 900 μL Buffer A, after which 100 μL of Buffer B (10 mM EGTA, 800 mM NaCl, 1% SOC, and 2% SDS) was added. The lysate was then split between two 1.5 mL Eppendorf tubes, and each lysate was sonicated for 5 cycles at 30% amplitude for 15 seconds on and 30 seconds off in an ice water bath, using a Branson SLPe Digital Sonifier and 1/8-inch probe tip. Sonicated lysates were then spun at 14,000*g* for 10 minutes, and the supernatant was transferred to a new 1.5 mL tube and glycerol added to 5% final concentration.

For IPs, 25 μg of chromatin was added to each tube to which 1 μg of rabbit anti-GATA2 (19) or rabbit IgG (Jackson ImmunoResearch) control was added, followed by RIPA buffer (MilliporeSigma) up to 1 mL. For input, 2.5 μg of chromatin was added and the antibody was omitted. The tubes were then rotated overnight at 4°C. The next day, 30 μL of protein G magnetic beads (New England Biolabs) were washed with RIPA buffer and then split equally across IgG and GATA2 tubes, which were then rotated for 4 hours at 4°C. Using a magnetic tube rack, the beads were then washed 3 times with 1 mL of RIPA buffer, 2 times with 1 mL of RIPA buffer + 300 mM NaCl, 2 times with 1 mL of LiCl buffer (250 mM LiCl, 0.5% NP-40, 0.5% DOC), and 1 time with 1 mL TE buffer (10 mM Tris pH 8.0, 1 mM EDTA, 50 mM NaCl). Beads then received 200 μL of ChIP Elution Buffer, and tubes were heated overnight at 65°C shaking at 800 rpm with 30 seconds on and 10 seconds off. Using a magnetic rack, the supernatant was then collected and 20 μg RNase A was added followed by 1 hour incubation at 37°C. The samples then received 20 μg Proteinase K and were incubated for 55°C for 2 hours, followed by cleanup using Qiagen PCR cleanup kit and elution into 50 μL EB buffer.

Purified immunoprecipitated and input DNA was submitted to the University of Wisconsin–Madison Biotechnology Center. DNA concentration and sizing were verified using the Qubit dsDNA HS Assay Kit (Invitrogen) and Agilent High Sensitivity D1000 ScreenTape (Agilent Technologies Inc.), respectively. Samples were prepared using NEBNext Ultra II DNA Library Prep Kit for Illumina (New England Biolabs) with minor modifications from the manufacturer’s protocol. Quality and quantity of the finished libraries were assessed using Agilent D1000 ScreenTape and Qubit dsDNA HS Assay Kit, respectively. Libraries were standardized to 2 nM. Paired end, 150 bp sequencing was performed using the Illumina NovaSeq X Plus (Illumina).

ENCODE ChIP-Seq pipeline (https://www.encodeproject.org/chip-seq/transcription_factor/) was used to align sequencing reads to human genome (hg38), call peaks, and generate signals. Protein-coding transcripts and long noncoding RNAs from GENCODE basic annotation (v38) were used to define exons and introns. Proximal promoter is defined as 1 kb upstream of a gene and distal promoter is defined as 4 kb upstream of a proximal promoter. All the rest are defined as intergenic regions. The genomic locations of ChIP-Seq peaks are determined by peaks’ overlap with the 5 types of genomic regions. Motifs for motif analyses were derived from HOCOMOCO database version 11.

### Statistics.

Statistical analyses were performed in GraphPad PRISM v.10 or R Suite and unless otherwise stated a *P* < 0.05 was considered significant. Overall survival and cancer-related survival was calculated as the time from diagnosis to death or time from diagnosis to cancer-related death, respectively, with surviving patients censored at the point of last follow-up. Recurrence-free survival was calculated as the time from diagnosis to recurrence, with nonrecurring patients censored at the point of last follow-up. Kaplan-Meier was used to estimate the median overall survival with a supporting 95% CI. Cox proportional hazard models were used to determine if survival associated with GATA2 percentage. The 2-tailed Student’s *t* test was used for comparisons across 2 groups, with Bonferroni correction for multiple comparisons when necessary. Fisher’s exact test was used for the analysis of categorical data across groups. Pearson correlation coefficients were used to compare GATA2, GATA3, and GATA6 IHC. For analysis of publicly available RNA-Seq datasets, the following quantiles were used for analysis using equal quantiles: low, minimum–33%; med, 33%–66%; high,66%–max. Differential quantiles include: low, minimum–50%; med,50%–75%; high, 75%–max. Single and multivariate analyses comparing the UW1 cohort to SEER data were modeled after a published analysis (44). The code and rationale utilized in that analysis may be downloaded as an html file at https://uwmadison.box.com/v/PopulationValidation Statistical significance was *P* < 0.05 with no adjustment for multiple testing.

### Study approval.

This study was approved under University of Wisconsin – Madison IRBs #2017-0765 (PI McGregor) and #2018-1510 (PI McGregor). All tumor tissues represented residual material collected during routine clinical care and patient consent was waived. Cases were considered USC if the patient’s final pathologic diagnosis was USC, endometrial serous carcinoma, or uterine papillary serous carcinoma. Cases were not included if the pathologist “favored” USC or if the organ source of the serous tumor remained unclear at the time of diagnosis.

### Data availability statement.

The data generated in this study are available within the article and in the Supporting Data Values file. Anti-GATA2 ChIP-Seq data have been deposited to Gene Expression Omnibus (GEO; accession no. GSE277397). RNA-Seq data have been deposited to GEO with accession no. GSE264610.

## Author contributions

USP, TEG, EC, RWS, IMO, MVM, LB, RB, BKE, VLH, SMM, EHB, and DRM designed research studies. USP, TEG, ATP, TSP, PMMM, JM, JP, RWS, MVM, and DRM performed experiments. USP, TEG, ATP, EC, TSP, MS, PMMM, JM, JP, RWS, MVM, RB, DER, MAK, and DRM acquired data. USP, TEG, ATP, PL, TSP, MS, PMMM, JM, JP, RWS, IMO, MVM, VLH, and DRM analyzed data. PH, LB, SMM, RB, BKE, VLH, EHB, and DRM provided reagents. PH, LB, EHB, and DRM wrote the manuscript. USP and TEG contributed equally to this work. USP is listed first because they took a lead role in the final stages of this work, and order of authorship was mutually agreed upon by USP and TEG.

## Supplementary Material

Supplemental data

ICMJE disclosure forms

Unedited blot and gel images

Supporting data values

## Figures and Tables

**Table 1:**
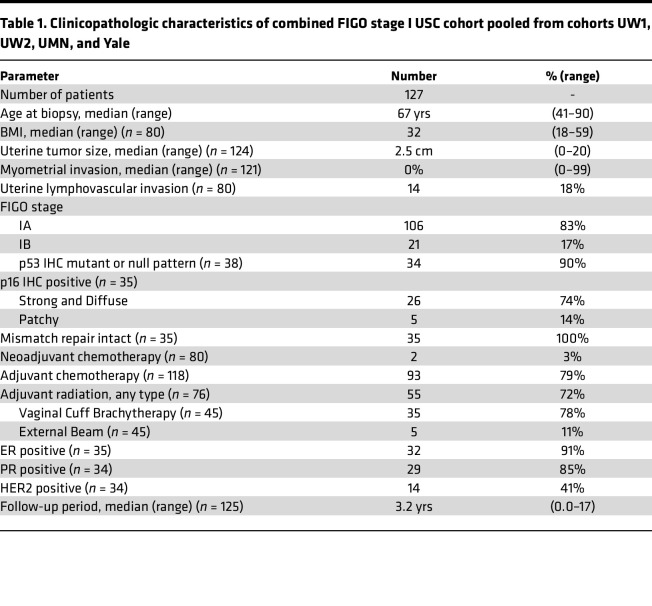
Clinicopathologic characteristics of combined FIGO stage I USC cohort pooled from cohorts UW1, UW2, UMN, and Yale

## Version 1 04/01/2025

In-Press Preview

## Version 2 05/08/2025

Electronic publication
