# Supplementary material for: Loss of GATA2 promotes invasion and predicts cancer recurrence and survival in uterine serous carcinoma
Source: JCI Insight. 2025 Apr 1;10(9):e187073. doi: 10.1172/jci.insight.187073 (PMC12128953; doi:10.1172/jci.insight.187073)

Supplemental Figure 1

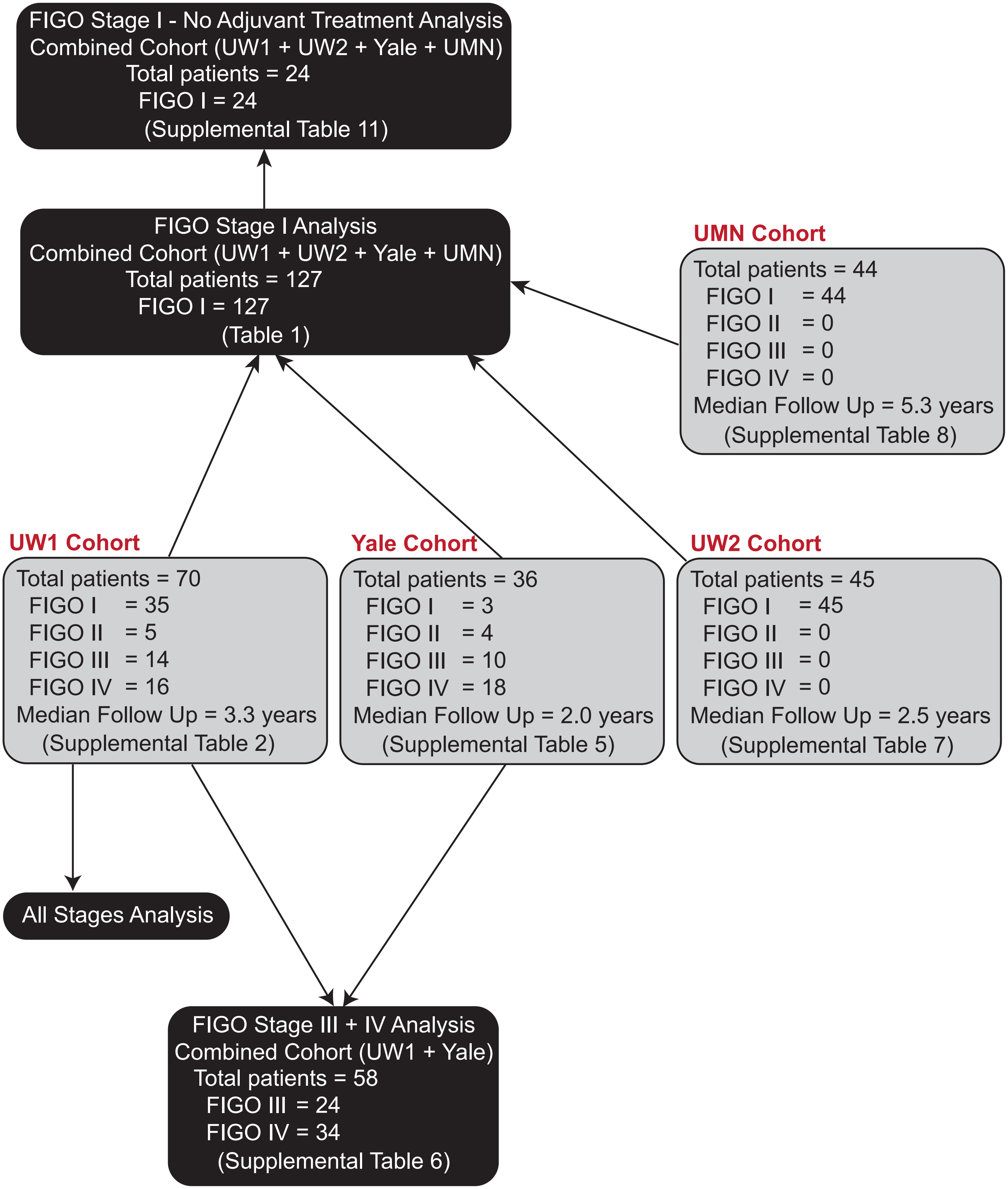

Supplemental Figure 2

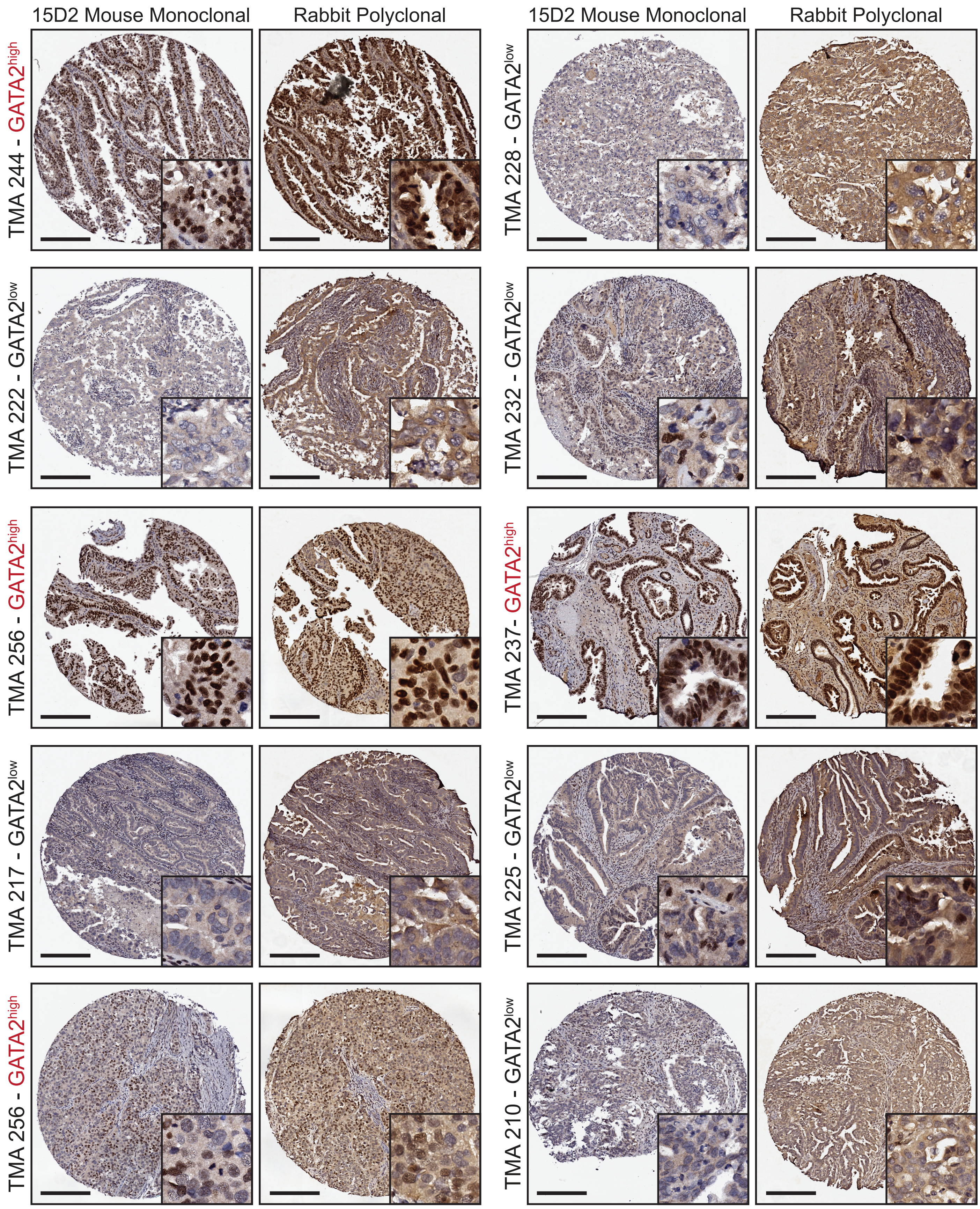

Supplemental Figure 3

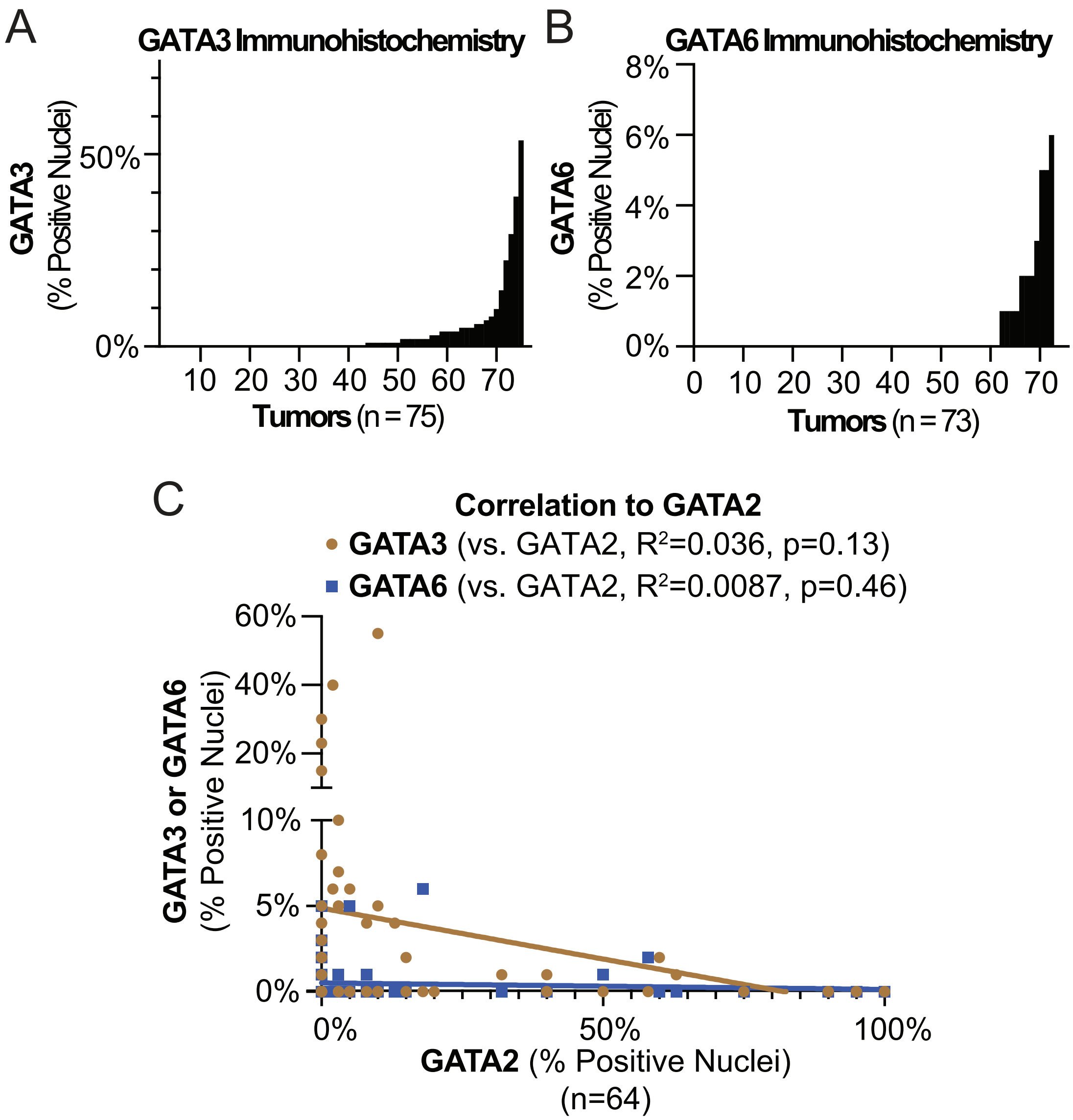

Supplemental Figure 4

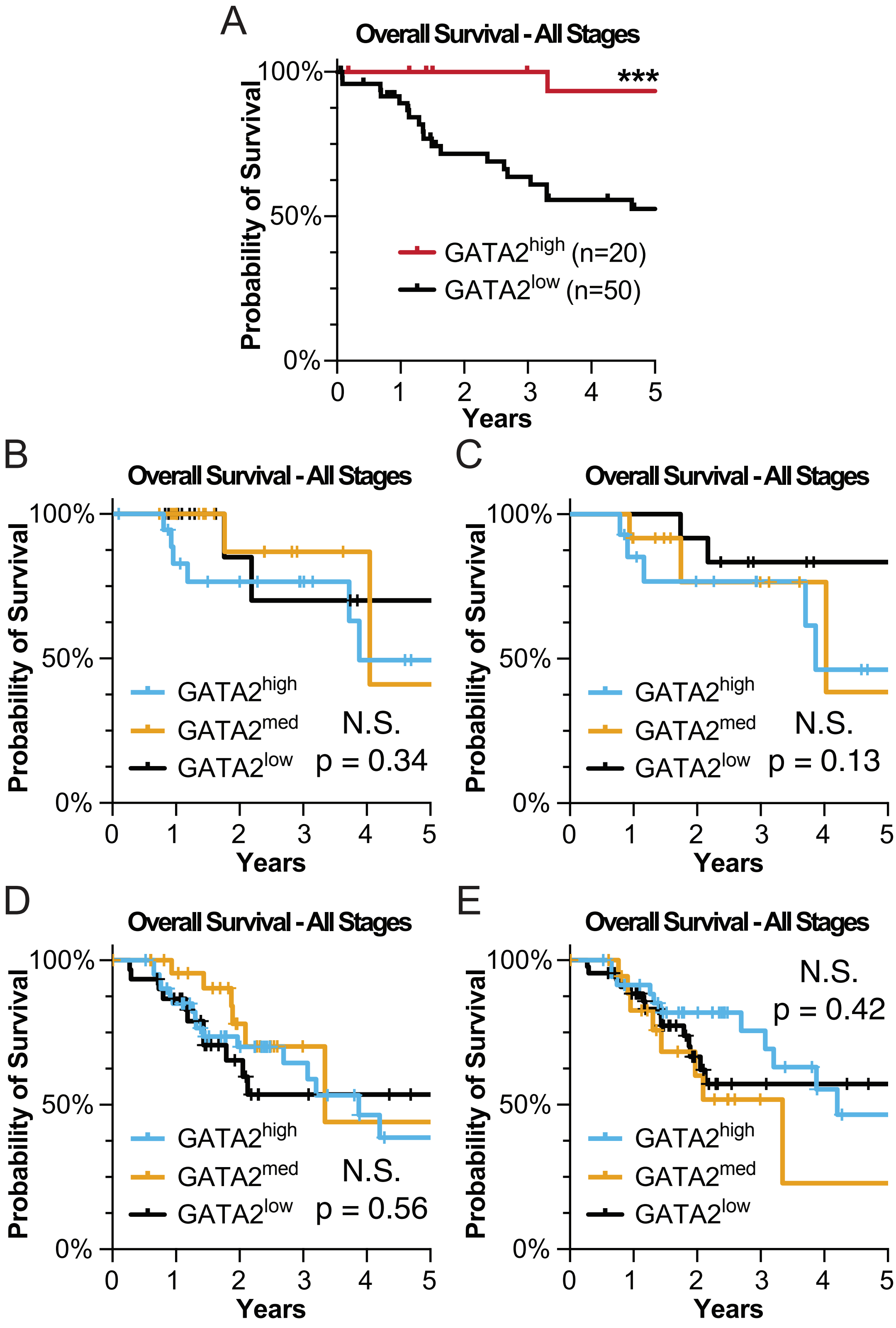

Supplemental Figure 5

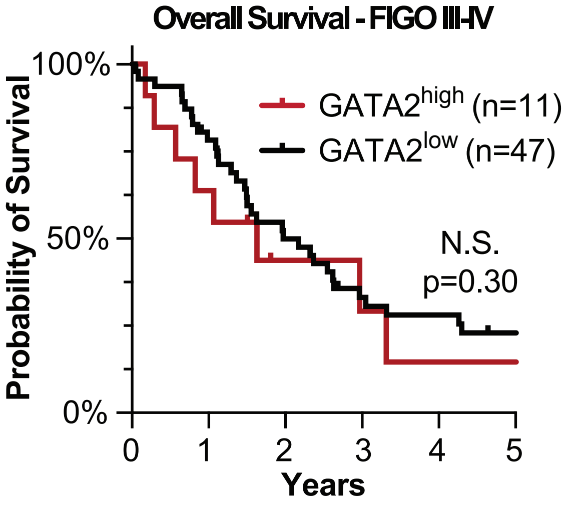

Supplemental Figure 6

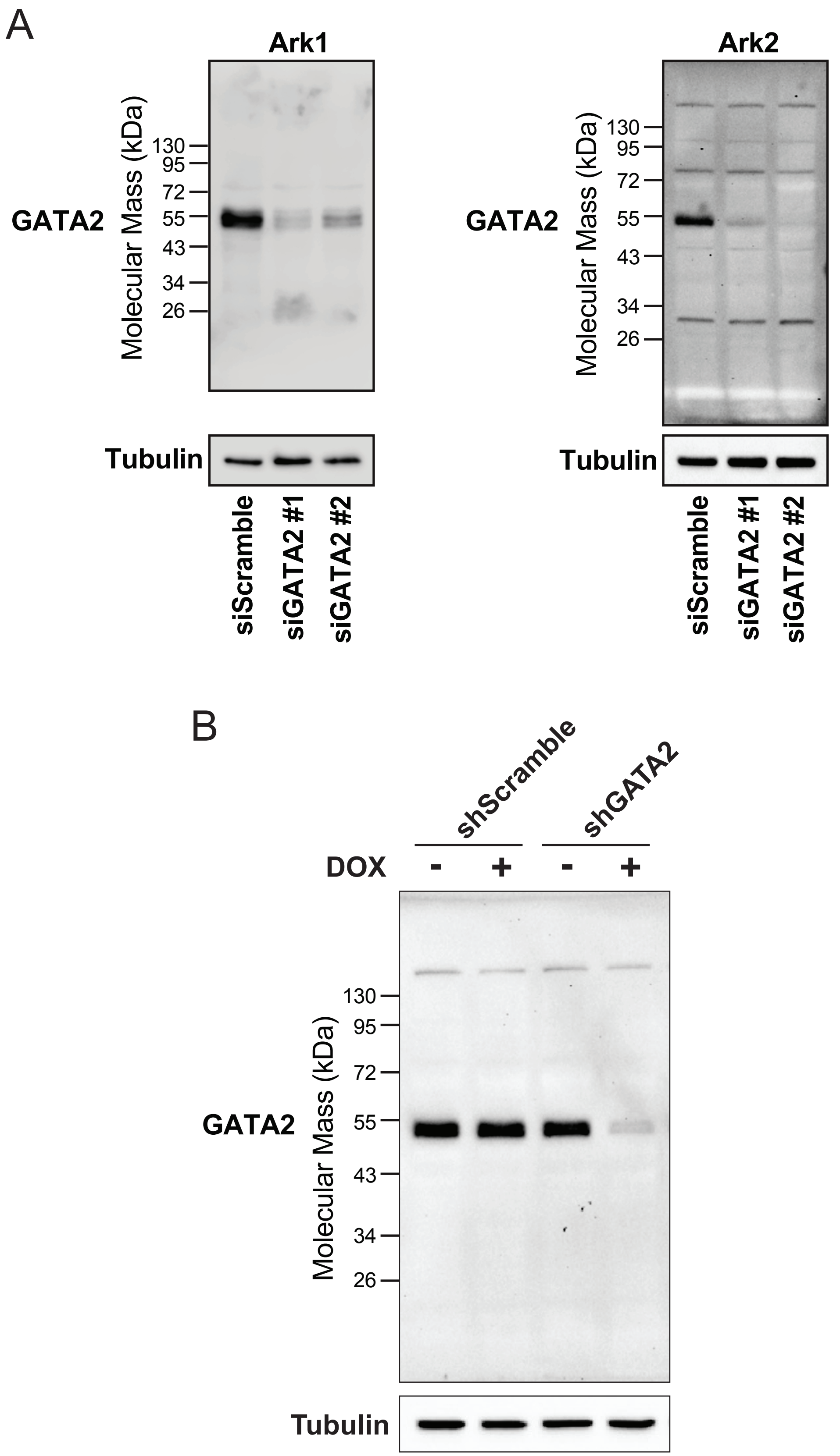

Supplemental Figure 7

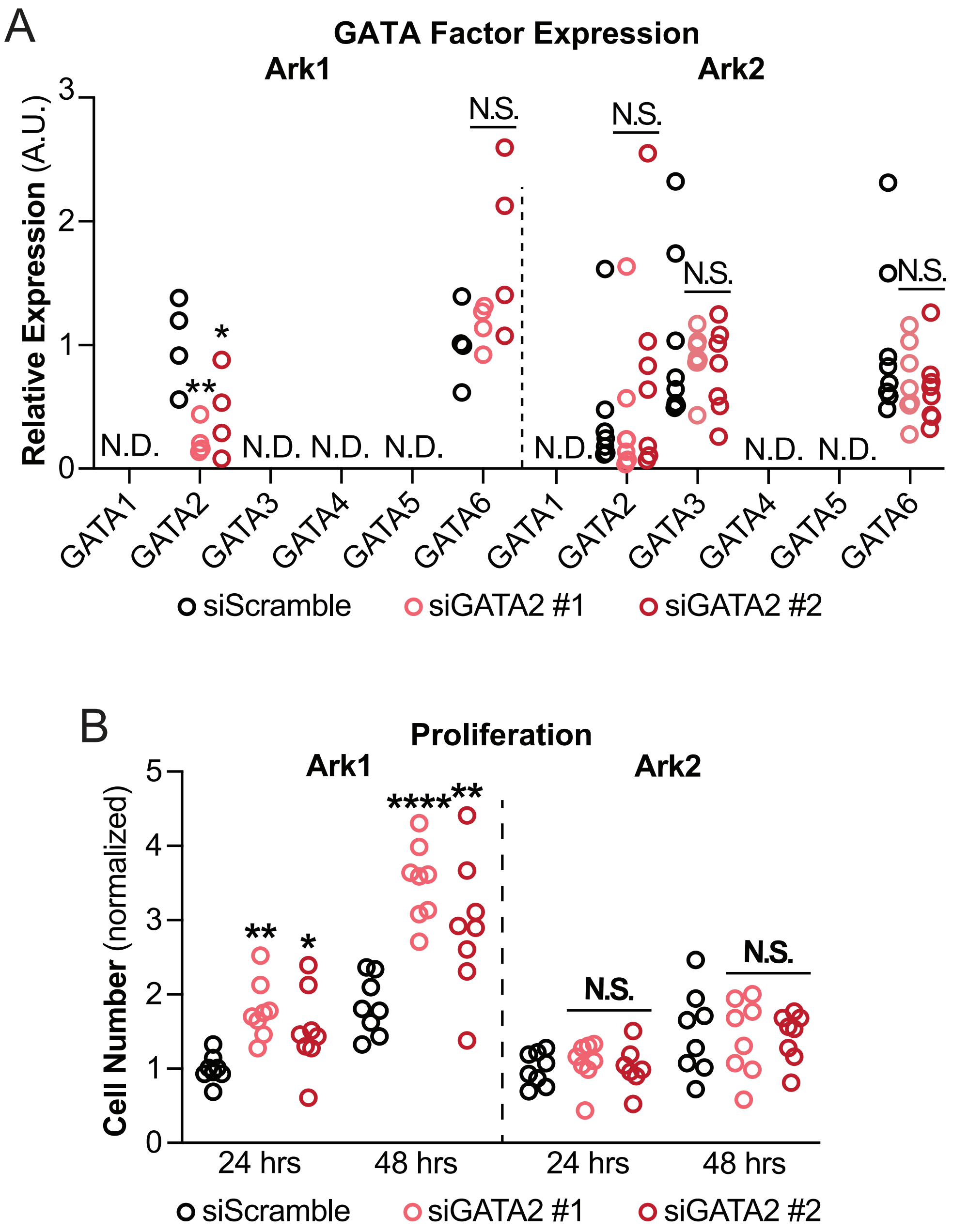

Supplemental Figure 8

A

Case	GATA2	Δ GATA2
1	10%	-9%
2	2%	-1%
3	10%	-9%
4	50%	-39%
5	10%	-9%
6	0%	0%
7	0%	0%
8	5%	-5%
9	3%	0%
10	0%	15%
11	5%	30%
12	0%	0%
13	32%	-28%
14	0%	0%
15	0%	0%
16	90%	-90%
17	0%	0%
18	0%	0%
19	3%	-3%
20	0%	2%
21	3%	3%
22	3%	11%

B

Omentum

Case	Δ GATA2
5	-9%
6	0%
7	0%
8	-5%
9	-3%
11	25%
14	0%

Periaortic Lymph Nodes

Case	Δ GATA2
1	-7%
2	0%
3	-9%
7	0%
10	38%
13	-27%
15	0%
18	0%
19	-3%
21	5%
22	12%

Abdomen/Pelvic Wall

Case	Δ GATA2
7	0%
8	-5%
9	-1%
10	0%

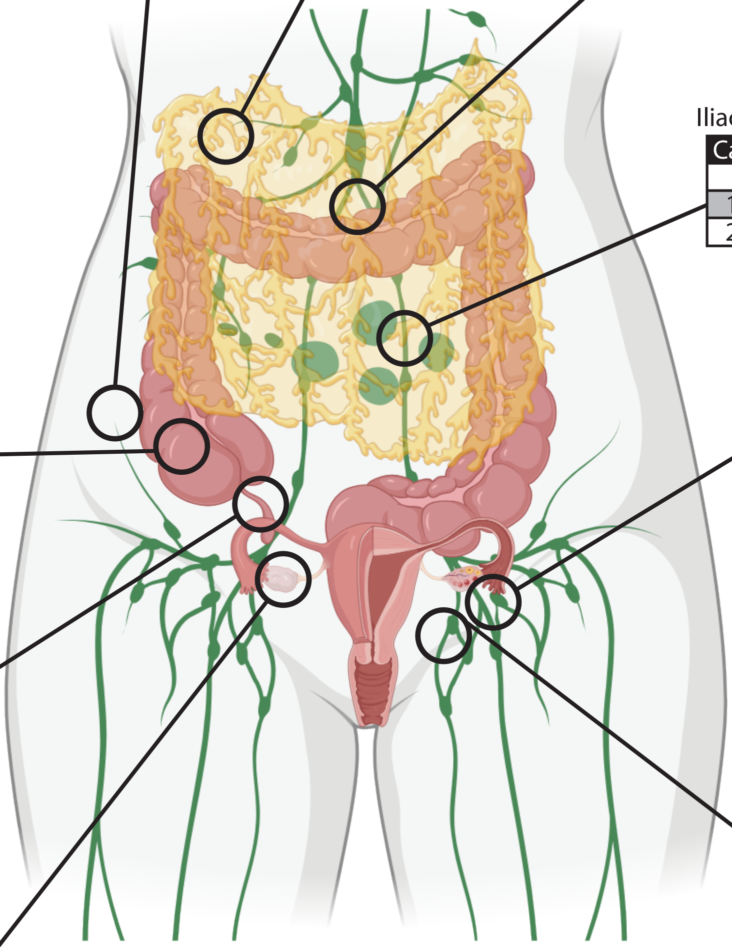

Iliac Lymph Nodes

Case	Δ GATA2
2	-1%
10	0%
22	7%

Bowel Wall

Case	Δ GATA2
5	-9%
6	0%
8	-5%
11	35%
16	-90%
20	5%

Obturator Lymph Nodes

Case	Δ GATA2
1	-10%
2	-2%
22	22%

Appendix

Case	Δ GATA2
4	-46%
5	-7%
9	4%
20	0%

Pelvic Lymph Nodes

Case	Δ GATA2
1	-10%
10	0%
12	0%
13	-28%
17	0%
18	0%
21	1%
22	2%

Ovary

Case	Δ GATA2
1	+5%
6	0%
7	0%
8	-5%
20	2%

Supplemental Figure 9

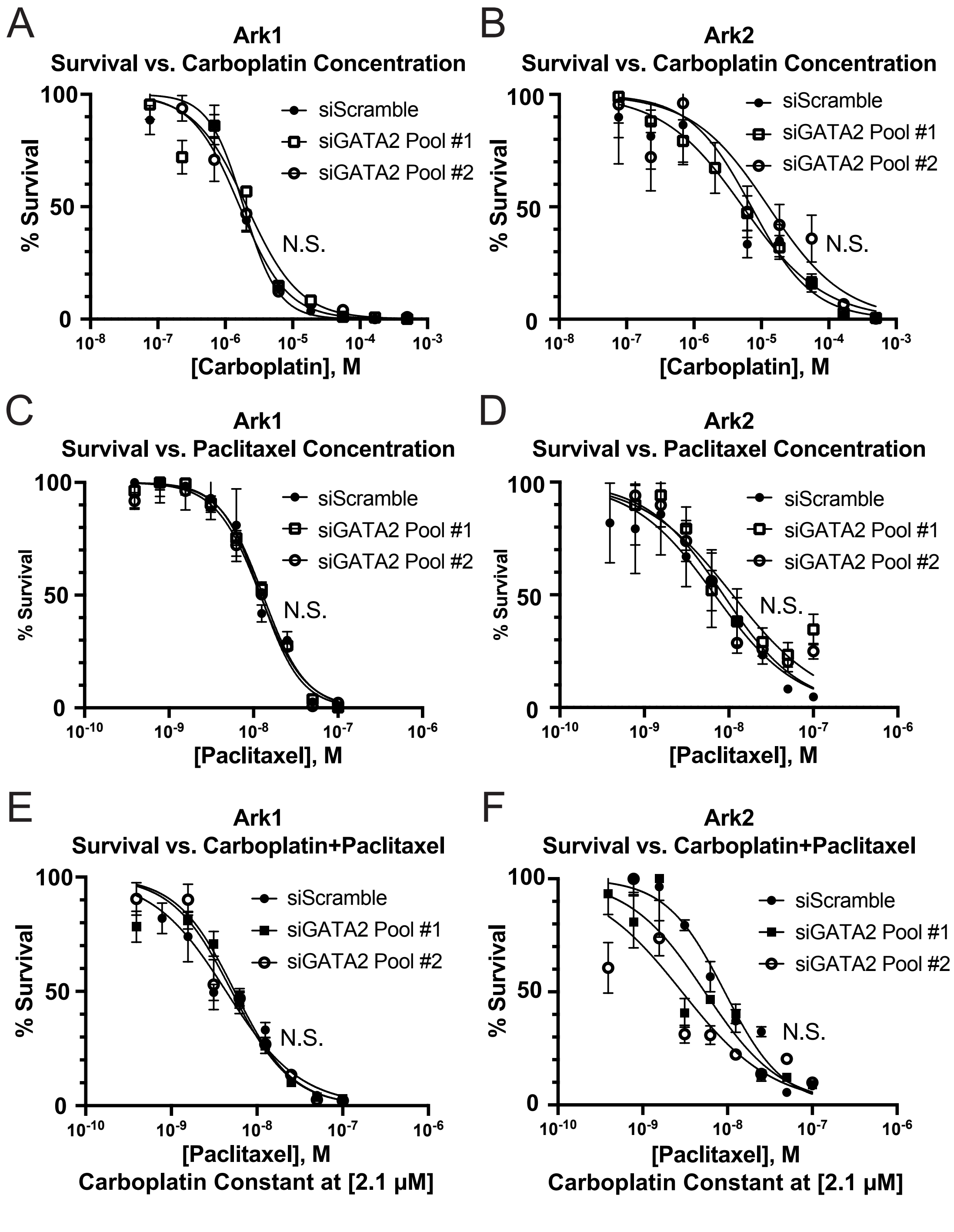

Supplemental Figure 10

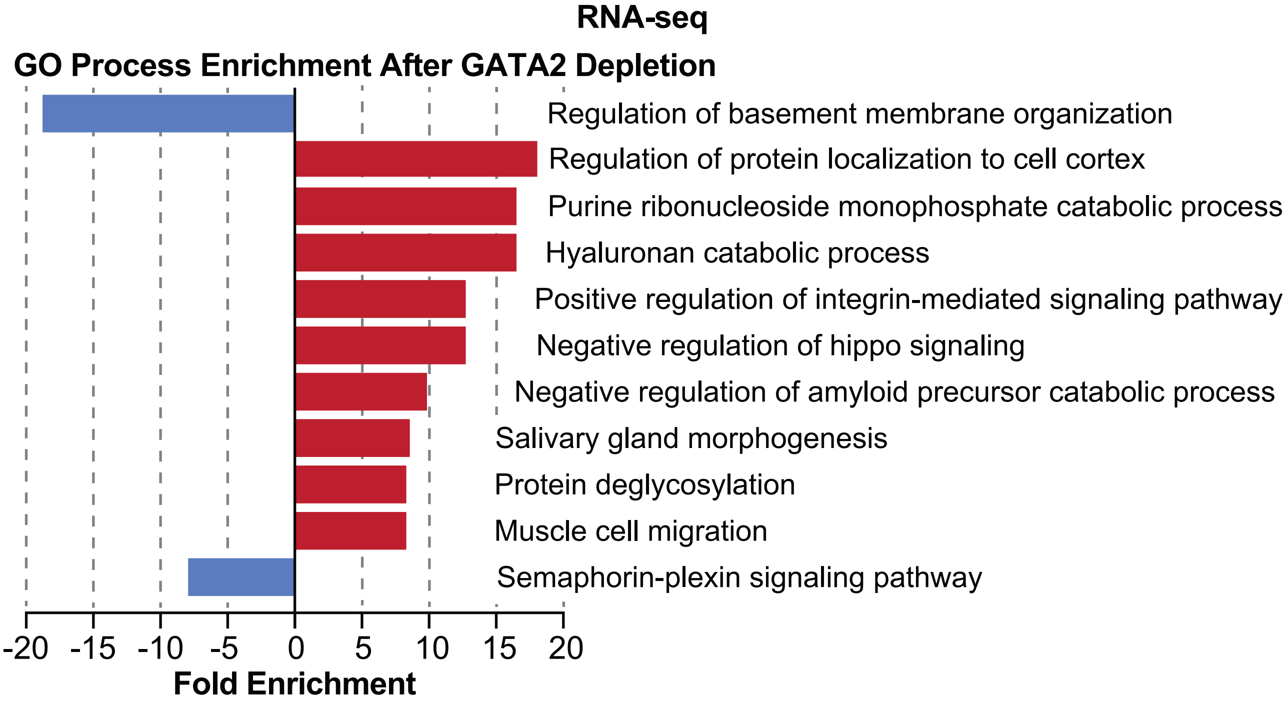

Supplemental Figure 11

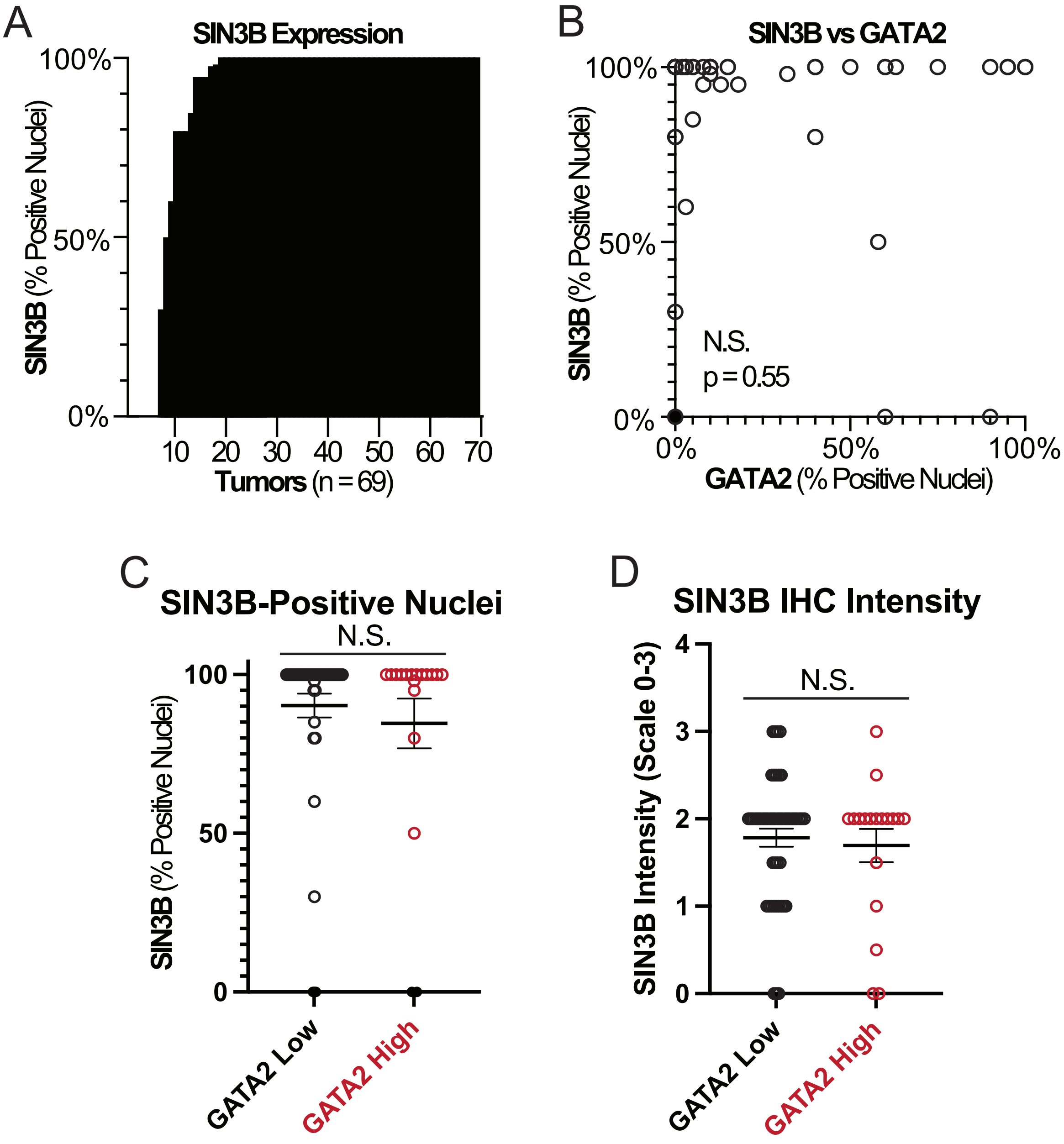

Supplement: Supplemental data [file jciinsight-10-187073-s210.pdf]
