# Supplementary material for: Loss of GATA2 promotes invasion and predicts cancer recurrence and survival in uterine serous carcinoma
Source: JCI Insight. 2025 Apr 1;10(9):e187073. doi: 10.1172/jci.insight.187073 (PMC12128953; doi:10.1172/jci.insight.187073)

Figure 3B

Ark1

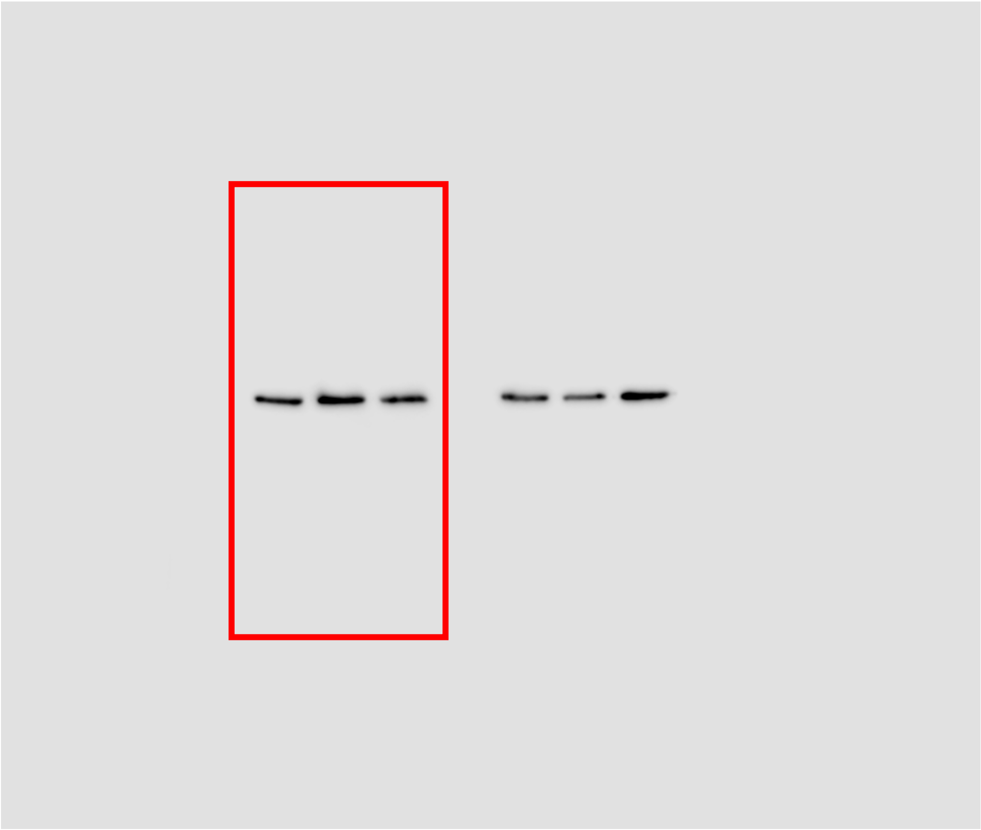

Anti-Tubulin

Note: Lanes from figure fall within the red box added by the author. The cropped lanes on the end represent an alternative cell line.

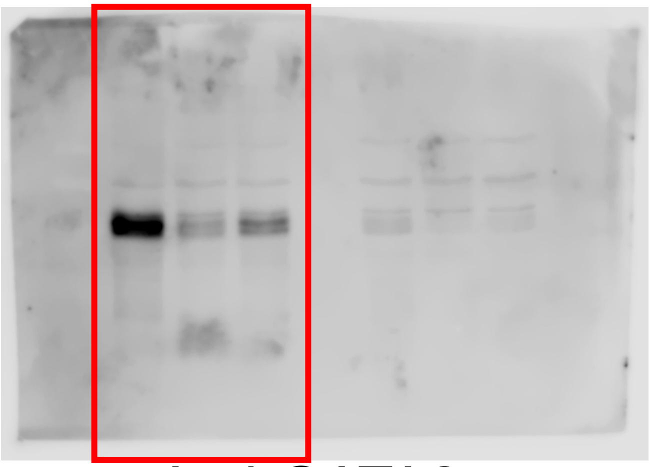

Anti-GATA2

Note: Lanes from figure fall within the red box added by the author. The cropped lanes on the end represent an alternative cell line.

Figure 3B (continued)

Ark2

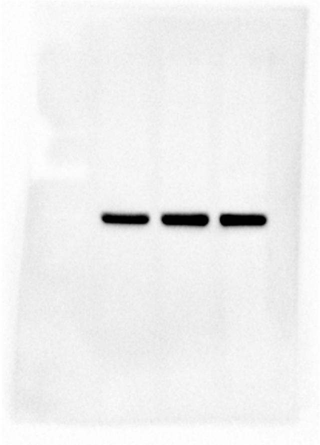

Anti-Tubulin

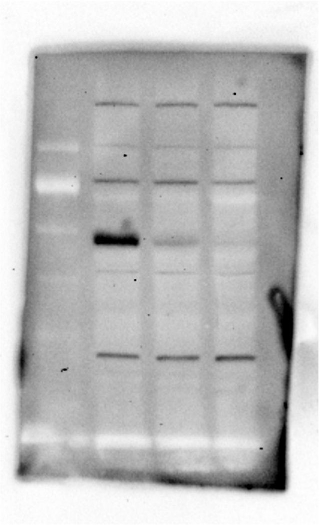

Anti-GATA2

Figure 3C

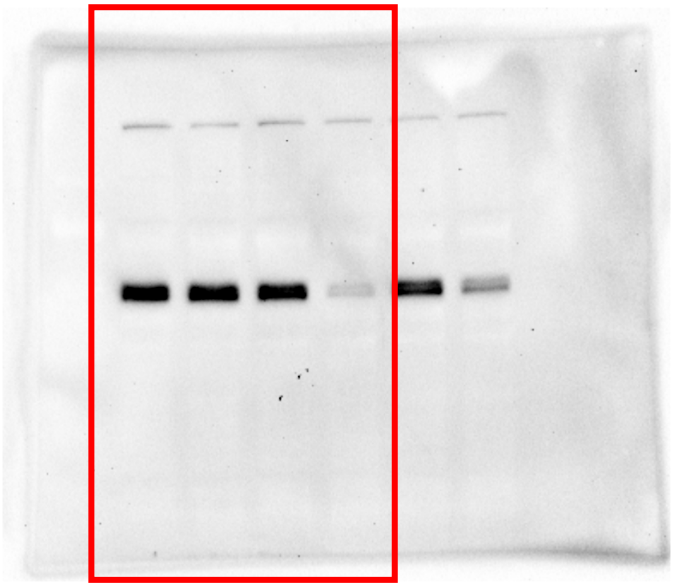

Anti-GATA2

Note: Lanes from figure fall within the red box added by the author. The cropped lanes on the end represent an alternative anti-GATA2 shRNA which also depletes but was not featured in the manuscript.

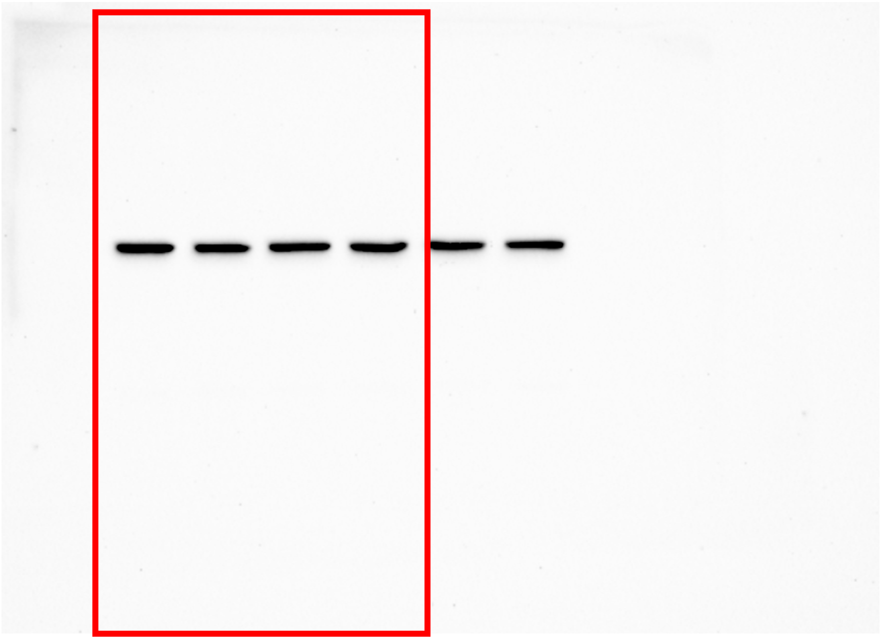

Anti-Tubulin

Note: Lanes from figure fall within the red box added by the author. The cropped lanes on the end represent an alternative anti-GATA2 shRNA which also depletes but was not featured in the manuscript.

Figure 4F

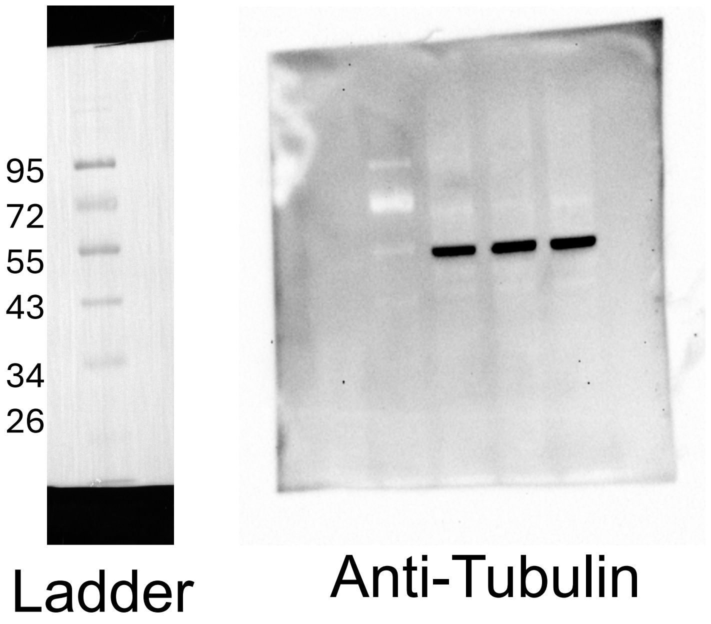
95
72
55
43
34
26
Ladder
Anti-Tubulin

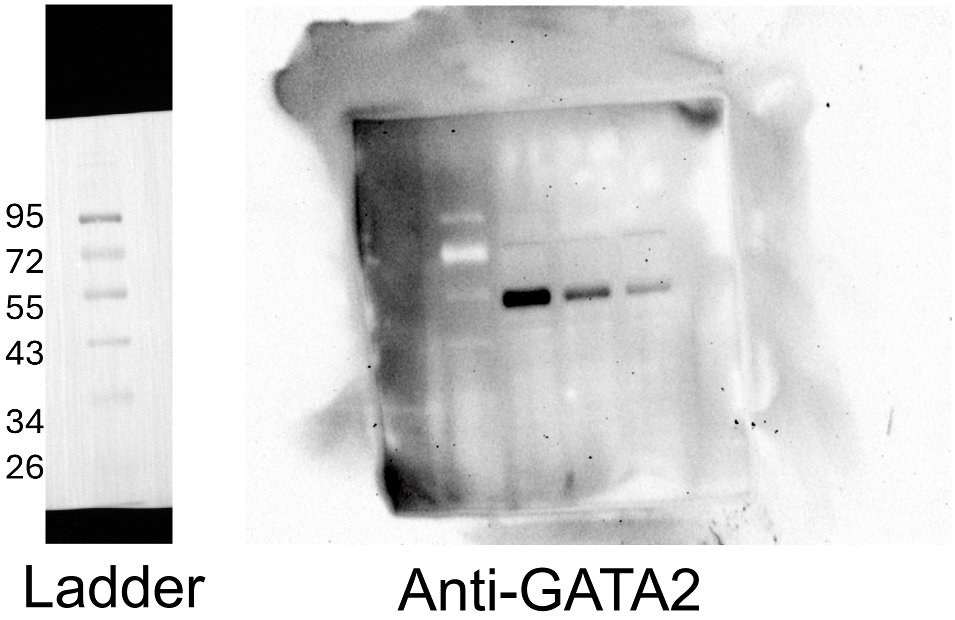
95
72
55
43
34
26
Ladder
Anti-GATA2

Figure 4F (continued)

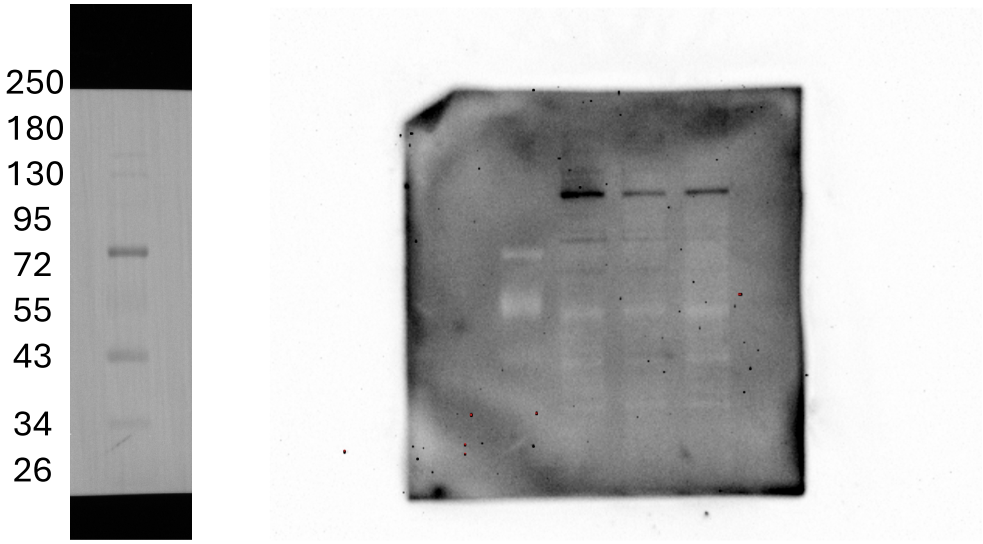

Ladder

Anti-SIN3B

Figure 4G

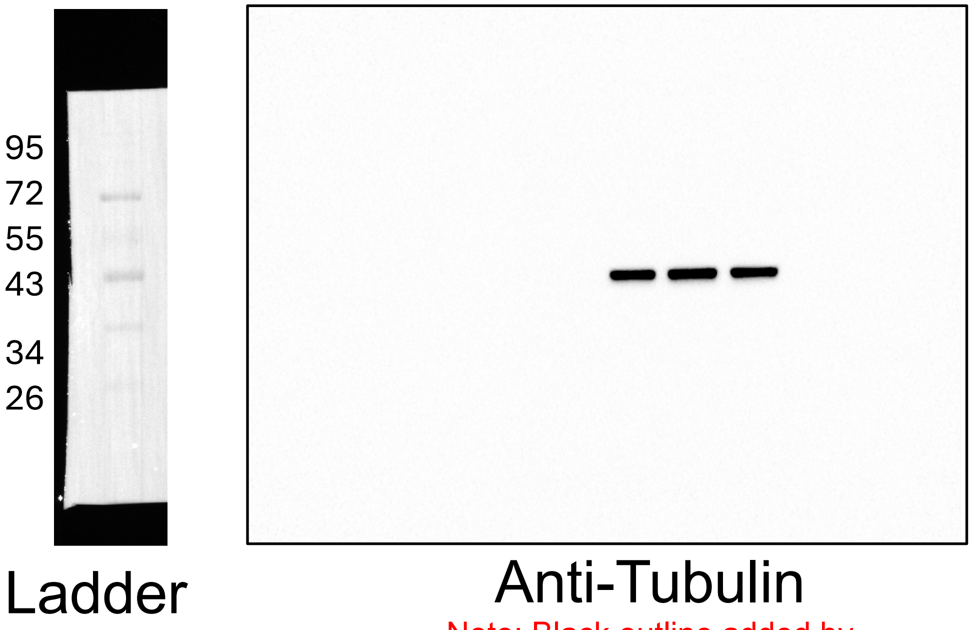

Note: Black outline added by author to demonstrate border of image.

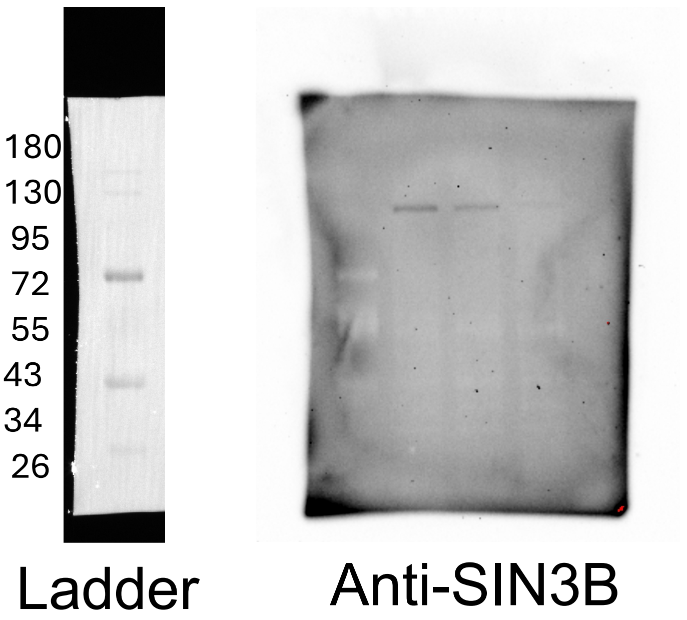

Supplement: Unedited blot and gel images [file jciinsight-10-187073-s212.pdf]
